# Volatilomic Analysis in Peel, Pulp and Seed of Hass Avocado ( *Persea americana* Mill.) From the Northern Subregion of Caldas by Gas Chromatography With Mass Spectrometry

**DOI:** 10.1002/fsn3.70489

**Published:** 2025-07-07

**Authors:** Juan Pablo Betancourt Arango, Alejandro Patiño Ospina, Jhon Alexander Fiscal Ladino, Gonzalo Taborda Ocampo

**Affiliations:** ^1^ Grupo de Investigación en Cromatografía y Técnicas Afines Universidad de Caldas Caldas Colombia

**Keywords:** GC–MS, Hass avocado, HS‐SPME, metabolic pathways, VOCs, volatilomics

## Abstract

Volatilomics, a subfield of metabolomics, is crucial for studying food quality and safety, ecological interactions, and natural product discovery. It allows characterizing biological matrices to identify secondary metabolites with biological potential. This study aimed to characterize VOCs present in the peel, pulp, and seed of Hass avocado and to reconstruct their associated metabolic pathways using HS‐SPME‐GC–MS. Hass avocado samples from the northern sub‐region of Caldas and commercial sources were analyzed. A 3D model of the fruit was used to define optimal sampling points. VOCs were extracted using HS‐SPME, separated, and identified by GC–MS, and compared against spectral libraries for compound confirmation. A metabolic enrichment analysis was performed using the hypergeometric test and betweenness centrality to identify key biosynthetic pathways. A total of 87 VOCs were identified, with the seed showing the greatest diversity, dominated by terpenes. Metabolites such as bicyclogermacene, β‐pinene, dodec‐(5Z)‐enyl acetate, cis‐β‐guaiene, α‐cadinene and δ‐cadinene had the greatest influence on the variability between samples. Sensory profiles related VOCs to aromatic traits: peel herbaceous, woody and sweet notes, pulp fatty and green characteristics, seed woody and medicinal attributes. Metabolic enrichment analysis identified several key pathways involved in volatile compound biosynthesis, including sesquiterpenoid and triterpenoid formation, glycolysis, pyruvate metabolism, and fatty acid synthesis, as well as the biosynthesis of cutin, suberin, and waxes. Additional contributions to the complexity of the Hass avocado volatilome were observed from pathways such as fatty acid elongation, GPI‐anchor biosynthesis, and fatty acid degradation. The peel, pulp, and seed of Hass avocado display distinct VOC profiles, suggesting tissue‐specific metabolic functions. Terpenes predominate in the seed, esters and aldehydes in the pulp, and oxygenated terpenes in the peel. These findings offer insight into the biochemical basis of avocado aroma and its relevance for food quality, defense mechanisms, and potential industrial applications.

## Graphical abstract

The volatilome of Hass avocado peel, pulp, and seed was characterized using HS‐SPME‐GC–MS, revealing 87 VOCs with tissue‐specific profiles. Terpenes dominated the seed, while esters and aldehydes were more abundant in the pulp, and oxygenated terpenes were prominent in the peel. Metabolic enrichment analysis highlighted key biosynthetic pathways including sesquiterpenoid/triterpenoid biosynthesis, glycolysis, and fatty acid metabolism, reflecting distinct biochemical roles across fruit tissues.

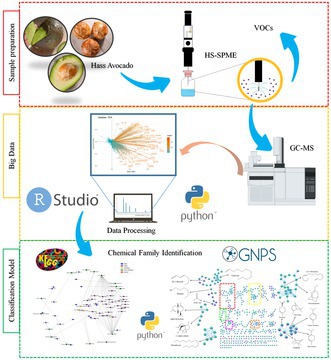

## 1 Introduction

In the study of Hass avocado ( *Persea americana* Mill.), a species of Mexican origin, more than two million tons are produced annually, with growth and development influenced by environmental conditions (Campuzano‐Granados and Cruz‐López 2021; Quiceno‐Rico et al. 2020). Despite its global importance, the aroma chemistry of Hass avocado remains underexplored, even though aroma plays a crucial role in consumer acceptance (Francisco et al. 2020). One of the key analytical methods for characterizing volatile organic compounds (VOCs) is headspace solid‐phase microextraction (HS‐SPME), which enables solvent‐free extraction and concentration while maintaining high sensitivity (Francisco et al. 2020).

Volatilomics, a branch of metabolomics, focuses on the study of VOCs—low‐molecular‐weight compounds emitted by biological systems (Betancourt‐Arango et al. 2025). These compounds play essential roles in ecological interactions, food quality, and plant physiology. In food science, volatilomics provides valuable insights into ripening, post‐harvest changes, and aroma profile characterization, using techniques such as HS‐SPME coupled with gas chromatography–mass spectrometry (GC–MS) (Betancourt‐Arango et al. 2024; Yang et al. 2020). This non‐invasive and cost‐effective approach allows for monitoring of ripening processes and quality control in fruit species without interfering with their natural maturation (Pinto et al. 2019).

HS‐SPME has been widely used for detecting trace compounds emitted by plants and environmental pollutants, making it a widely used technique in both in vitro and in vivo studies (Zhu et al. 2013). It has been applied to different plant tissues, including fruits, flowers, leaves, stems, roots, and seeds (Soledad et al. 2021). In the case of Hass avocado, previous studies have characterized its volatile profile, identifying compounds such as terpenes, aldehydes, and fatty acid derivatives (Abubakar et al. 2017). For instance, research on Hass avocado seed has identified terpenic compounds like estragole, isoestragole, alpha‐cubebene, and alpha‐caryophyllene, along with aldehydes such as palmitaldehyde and fatty acids including linoleic acid methyl ester (Abubakar et al. 2017). Among these, triterpenoids have demonstrated anticancer activity (Xagoraris et al. 2022).

Similarly, VOCs present in avocado oil have been analyzed in relation to biological activity and product quality. The volatile composition varies according to the fruit's ripening stage, with 44 identified terpenic compounds, along with non‐terpenic hydrocarbons (Estrada 2018). From a biological perspective, VOCs serve as mechanisms of attraction and defense against herbivorous phytopathogens. They are present in branches, leaves, and fruits, with hydrocarbons, aldehydes, and terpenes being the most commonly identified compounds (Mostafa et al. 2022). Moreover, VOCs contribute to pollination and seed dispersal, including terpenoids, phenylpropanoids, benzenoids, and fatty acid derivatives (Castañeda‐Antonio et al. 2015).

Most studies on Hass avocado have primarily focused on polyunsaturated fatty acids (oleic, linoleic, and palmitoleic acids) and their nutritional benefits. However, limited research has been conducted on the influence of genetic and geographical variations on VOC composition. A recent study identified 26 VOCs in avocado leaves, including monoterpenoids, sesquiterpenes, and phenylpropanoids (Astudillo‐Ordóñez and Rodríguez 2018). Comparable metabolites have also been identified in the Drymifolia variety, including phenolic compounds, fatty acids, and acetogenins, which have been associated with antioxidant, anti‐inflammatory, and anticancer activities (Lara‐García et al. 2021; Salazar‐López et al. 2020).

The molecular families of bioactive compounds in avocado include phenolic acids (hydroxycinnamic and hydroxybenzoic acids) (Lyu et al. 2023), flavonoids, proanthocyanidins, acetogenins, phytosterols, carotenoids, and alkaloids, highlighting the fruit's functional diversity (Ali et al. 2020). Studies have reported key VOCs such as acetaldehyde, hexanal, (E)‐2‐hexenal, limonene, and beta‐caryophyllene, as well as the presence of 1‐octen‐3‐one and (E,E)‐2,4‐nonadienal (Hausch et al. 2020). Efficient VOC extraction depends on optimizing variables such as extraction time and temperature, which significantly impact the volatile profile (Lopez‐Vega et al. 2021). Additionally, the physicochemical properties of Hass avocado include chlorophyll, lutein, beta‐sitosterol, alpha‐tocopherol, and oleic acid in the pulp, while anthocyanins, glycosylated quercetin, and mannoheptulose have been identified in the peel (Ramos‐Aguilar et al. 2021).

The integration of metabolomics and proteomics has provided insights into glycolytic reduction and alternative ripening pathways, revealing substantial differences in Hass avocado samples based on heat treatment exposure (Gavicho‐Uarrota et al. 2019). These findings have led to improved post‐harvest homogeneity in ripening through the stabilization of soluble sugars and oxidative stress‐related enzymes (Liu et al. 2021). Additionally, volatilomics has been applied to analyze avocado storage conditions, where gas chromatography‐ion mobility spectrometry (GC‐IMS) and principal component analysis (PCA) have been used to assess ripening‐induced VOC changes, facilitating predictions about fruit maturity.

For Hass avocado, VOCs have been identified in leaves, flowers, and mesocarp, with 31 compounds detected in unripe fruits, including alpha‐copaene, beta‐copaene, and beta‐caryophyllene. VOC differentiation has been observed between Hass and Fuerte varieties, where estragole was more abundant in the latter. In this way, metabolomics, and especially volatilomics, allows research on food quality and safety, as well as inducing the development of exploratory and bioprospective studies for the discovery of new natural products and evaluations of chemo‐ecological interactions (Selamat et al. 2021; Vargas‐Abasolo et al. 2022). Another aspect that has been studied is the effect of grafting on VOC composition, where the use of rootstocks has been shown to influence the production of biogenic VOCs. Through HS‐SPME‐GC–MS, grafted Hass avocado plants have been found to produce a variety of monoterpenes such as beta‐pinene, cumene, 3‐carene, R‐limonene, and (Z)‐beta‐ocimene, highlighting the influence or rootstocks on the volatile profile (Ceballos and Rioja 2019).

The volatile profile of avocado peel includes a diverse array of compounds, notably terpenoids such as linalool, β‐caryophyllene, α‐terpinene, and limonene. In addition, carboxylic acid derivatives (e.g., acetic and butyric acids), aliphatic hydrocarbons (such as hexane and octane), aromatic compounds (including benzaldehyde and phenylethanol), steroid‐like molecules (such as ethyl stearate), and nitrogen‐containing volatiles (e.g., indole and pyridine) have been reported. Furthermore, esters such as ethyl hexanoate, ethyl octanoate, and ethyl nonanoate have also been detected, contributing to the overall chemical complexity of the peel's volatilome. Considering the relevance of VOCs in food quality assessment, safety monitoring, and the discovery of novel natural products, this study investigates the volatile organic compounds comprising the volatilome of Hass avocado peel, pulp, and seed from the northern sub‐region of the Caldas department, employing HS‐SPME coupled with GC–MS for their extraction and identification.

## 2 Methodology

### 2.1 Sampling

The different samples of *Persea americana* Mill. were obtained from various local markets and sales points in Manizales, Caldas, Colombia. Additionally, other sample varieties were acquired through the Department of Agriculture of Caldas from the municipalities of Pacora, Aranzazu, and Salamina. Sample collection took place between April and May 2024, during the fruit's harvest season. The samples were selected based on size uniformity, ensuring the absence of physical damage or visible signs of disease. They were transported in sealed containers at approximately 5°C to inhibit metabolic activity and reduce respiration rate. Upon arrival at the laboratory, the samples were stored at controlled ambient temperature before processing. To ensure uniform ripening, all processed samples were collected at an intermediate ripening stage (level three), characterized by a soft‐textured, dark‐colored exocarp, while the mesocarp (pulp) remained soft yet firm when pressed. The endocarp (inner lining) and seed were visually inspected to confirm the absence of alterations or anomalies. This traceability approach enabled the standardization of external conditions that could influence metabolite expression, minimizing the impact of uncontrolled ripening factors. Samples were selected through a completely randomized sampling process. Once obtained, they were separated into exocarp, mesocarp, and endocarp.

### 2.2 VOC Extraction Procedure

VOCs were extracted using headspace solid‐phase microextraction (HS‐SPME) with a gray fiber composed of polydimethylsiloxane, divinylbenzene, and carboxen (PDMS/DVB/CARB, 50/30 μm). For each extraction, 2.5 g of Hass avocado peel, pulp, or seed was weighed and mixed with 0.4 g of NaCl to enhance the salting‐out effect, followed by homogenization. An ultrasound pre‐treatment was applied for 20 min to improve analyte release from the matrix (Kilic‐buyukkurt 2021). Extraction was performed at 52°C for 40 min, followed by desorption at 250°C for 15 min in the GC–MS injection port.

The selection of HS‐SPME‐GC–MS was based on its demonstrated efficiency, sensitivity, and reproducibility for profiling complex plant matrices. Compared to other VOC extraction techniques—such as simultaneous distillation‐extraction (SDE), stir bar sorptive extraction (SBSE), and solvent‐assisted flavor evaporation (SAFE) (Soledad et al. 2021)—HS‐SPME is solvent‐free, non‐destructive, and requires minimal sample preparation, which makes it ideal for preserving native volatile profiles, especially in lipid‐rich matrices like avocado. Additionally, the fiber used (DVB/CAR/PDMS) offers broad analyte coverage for low‐molecular‐weight compounds including terpenes, aldehydes, esters, and alcohols (Zhang et al. 2021). The main limitations of HS‐SPME include its semi‐quantitative nature, dependency on analyte‐fiber affinity, and limited extraction capacity for high‐boiling or strongly matrix‐bound compounds. Nevertheless, for exploratory volatilomics, these limitations are acceptable when coupled with rigorous spectral validation.

The extraction conditions were determined through preliminary optimization trials conducted in our laboratory, aiming to maximize metabolite recovery while minimizing matrix degradation. These essays are documented in the master's thesis entitled: Volatilomic analysis of Hass avocado ( *Persea americana* Mill. cv.) (peel, pulp and seed) from the Northern sub‐region of Caldas for the identification of pesticides and potential biomarkers of toxicity, available at the institutional repository (access restricted until 2026): https://repositorio.ucaldas.edu.co/entities/publication/6f992d35‐b037‐41c4‐8dfe‐196e6a20f90b. These parameters are also consistent with prior HS‐SPME studies in avocado, which report optimal extraction ranges between 50°C–60°C and 30–45 min. Each sample—whether of commercial origin or collected from the northern subregion of Caldas—was processed in triplicate to ensure reproducibility of VOC extraction, separation, and identification (Xagoraris et al. 2022).

### 2.3 GC—MS Analysis

A Shimadzu Thermo Scientific TRACE QP2010 Plus gas chromatograph (GC) coupled with a mass spectrometry (MS) detector was used for the separation and detection of VOCs in Hass avocado. The analysis was performed using a ZB‐5 column (30 m × 0.25 mm ID × 0.25 μm film thickness). A temperature gradient was applied to achieve optimal compound separation. The initial temperature was set at 45°C for 2 min, followed by an increase of 3°C/min until reaching 100°C, where it was held for 1 min. The temperature was then raised to 300°C at a rate of 10°C/min and held for 2 min, resulting in a total analysis time of 43.33 min. The injection was performed in solid‐phase microextraction (SPME) mode using helium as the carrier gas. The injection port was maintained at 250°C, while the detector operated at 290°C. Detection was conducted in scan mode under electron impact ionization (EI) at 70 eV.

### 2.4 Identification of the Volatilome

For the identification of volatile organic compounds (VOCs) obtained through HS‐SPME‐GC–MS, Solution Version 4.30 software from the Shimadzu Thermo Scientific TRACE QP2010 Plus system was used. Compound identification was performed using the NIST14 library with an 80% similarity threshold, along with the NIST14S, ADAMS, ESSENTIAL OILS, and FFNSC (Flavor and Fragrance Natural and Synthetic Compounds) libraries, applying a 90% similarity threshold. Peak smoothing and area integration were performed using the software. Additionally, a series of alkanes was analyzed to determine Kovats retention indices (KI). To further validate compound identification, the data were processed through the Global Natural Products Social Molecular Networking (GNPS) database, allowing for peak deconvolution, GC–MS data identification, and the construction of a molecular network correlating the VOCs present in the peel, pulp, and seed of Hass avocado. For the final compound report, the following parameters were included: compound name, molecular formula, mass‐to‐charge ratio (m/z), boiling point (BP), retention time (RT), theoretical Kovats index (KI Teo), experimental Kovats index (KI Exp), similarity percentage (%), peak area, adduct, KEGG code, LipidMaps code, Human Metabolome Database (HMDB) reference code (Wishart et al. 2022), and error percentage (Δ ppm).

### 2.5 Molecular Mapping

In food matrices, metabolic flux analysis provides insights into the spatial distribution of compounds. In Hass avocado, metabolites are not homogeneously distributed but vary depending on their proximity to the peduncle, which connects the fruit to the plant's branch. To optimize the selection of sampling points and improve the interpretation of VOC distribution, molecular mapping was integrated into the analysis process. Technological advances have enabled precise mapping of the spatial coordinates of sampling points on the fruit's surface, facilitating a more detailed assessment of metabolite distribution. To achieve this, Blender software (https://www.blender.org/download/) (Aziyen and Ahmed 2020), was used to create a 3D model of the avocado, while MeshLab software (https://www.meshlab.net/) (Pardo‐Pérez 2018; Million and Kenkel 2020), was employed to establish the spatial coordinates of each sampling point. This integration of computational tools enhances the visualization and interpretation of metabolite distribution, allowing for a more systematic identification of metabolic patterns within the Hass avocado matrix.

### 2.6 Olfactometric Analysis

After the identification process, the Flavornet database (Arn and Acree 1998), was used to obtain olfactometric data on the compounds present in the peel, pulp, and seed of Hass avocado. The objective was to construct a theoretical olfactometric profile, describing the predominant aromas naturally emitted by each part of the fruit. This profile provides insight into the sensory attributes of Hass avocado by correlating its volatile composition with its perceived aromatic quality, offering a theoretical basis for assessing its sensory characteristics.

### 2.7 Quality Assurance and Quality Control

To ensure the reliability and accuracy of the results obtained in this study, a quality assurance (QA) and quality control (QC) protocol was implemented. This included the use of quality control samples, instrument performance verification, and data validation steps (Evans et al. 2020). A total of 38 quality control samples were analyzed. For gas chromatography (GC) analysis, quality controls were distributed as follows: 10 column quality controls (CQC) and 12 system quality controls (SQC) to monitor the instrument's response through solvent injections. Since VOC extraction was performed using HS‐SPME, an additional 13 fiber quality controls (FQC) were incorporated to verify injection quality and prevent potential memory effects due to compound retention on the fiber surface between samples. These quality controls enabled the evaluation of chromatographic system stability and background contamination within the column. CQC samples consisted of blank injections to monitor column performance and identify potential cross‐contaminations between samples. SQC samples, which contained only the solvent (acetone) used for column cleaning, were injected to detect and correct background noise, eliminate contaminants between volatilomic sample injections, and assess signal variation in the analytical instrument.

### 2.8 Multivariate Analysis

To further investigate the differences in VOC composition among Hass avocado samples from the northern sub‐region of the Caldas department, multivariate statistical analyses were conducted in RStudio. Both supervised and unsupervised techniques were applied to reduce dataset dimensionality and identify clustering patterns based on VOC profiles obtained via GC–MS. Principal Component Analysis (PCA) was employed to capture variability and reveal clustering trends among samples. Additionally, Hierarchical Cluster Analysis (HCA) was performed using the Euclidean distance method to classify samples according to their volatile composition. These analyses were conducted using the RStudio packages *timeSeries*, *reshape2*, *cluster*, *gplots*, *ggfortify*, *RColorBrewer*, *ggplot2*, *tidyverse*, *base*, *graphics*, *stats*, *fBasics*, and *pvclust*.

### 2.9 Metabolic Pathway

Once the metabolites comprising the volatilome were identified, their corresponding Human Metabolome Database (HMDB) and KEGG codes (Kanehisa et al. 2023), were retrieved to perform a metabolic pathway enrichment analysis using MetaboAnalyst (Chen et al. 2022; Chong et al. 2019). This analysis involved calculating *p*‐values and their −log(*p*) transformations to assess the statistical significance of the identified biochemical pathways. Additionally, the pathway analysis was complemented by a Python script designed to retrieve KEGG codes for the identified metabolites and generate an interaction network linking metabolites, enzymes, and associated biochemical pathways.

### 2.10 Molecular Mapping

**FIGURE 1:**
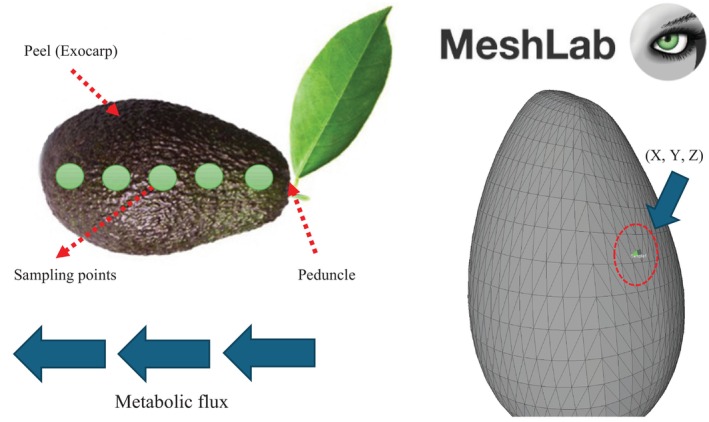
Molecular mapping: Identification of sampling point in 3D model for identification of spatial coordinates of sampling.

In Figure 1, multiple sampling points can be defined across the surface of the Hass avocado matrix. However, the highest metabolite concentration is found near the peduncle, where the fruit connects to the plant's branches. This distribution reflects the intrinsic biochemical and metabolic flow within the plant. To determine the sampling points, a 3D model of the Hass avocado was constructed using Blender. This model was then imported into MeshLab, where spatial coordinates X, Y, and Z (−4.32848, −3.38426, 11.9374) were established as the reference point for sampling in this study, located approximately at the midpoint of the fruit.

From this reference point in the 3D model, the necessary quantities were extracted for analysis in the peel, pulp, and seed. Additionally, this sampling point was used to identify different volatile metabolites within the matrix using HS‐SPME, followed by their separation and identification via GC–MS. This approach enables the correlation of identified metabolites with their specific location within the fruit, providing a fluxomic analysis perspective for Hass avocado (Emwas et al. 2022).

## 3 Results and Discussion

### 3.1 Volatilome Constituent Metabolites

Regarding the extraction methodology, once the sampling point was established, the identification of compounds was performed using GC–MS with a 90% similarity index based on the referenced libraries. Additionally, the mass‐to‐charge ratio (m/z) and retention time for each detected signal were determined. Compound confirmation was achieved by comparing theoretical and experimental mass fragmentation patterns, as well as utilizing the Kovats index (KI) in both its theoretical and experimental forms. The experimental KI was calculated by interpolating each signal within a series of alkanes (C_7_–C_40_), allowing for the determination of the percentage error for each identified compound.

**FIGURE 2:**
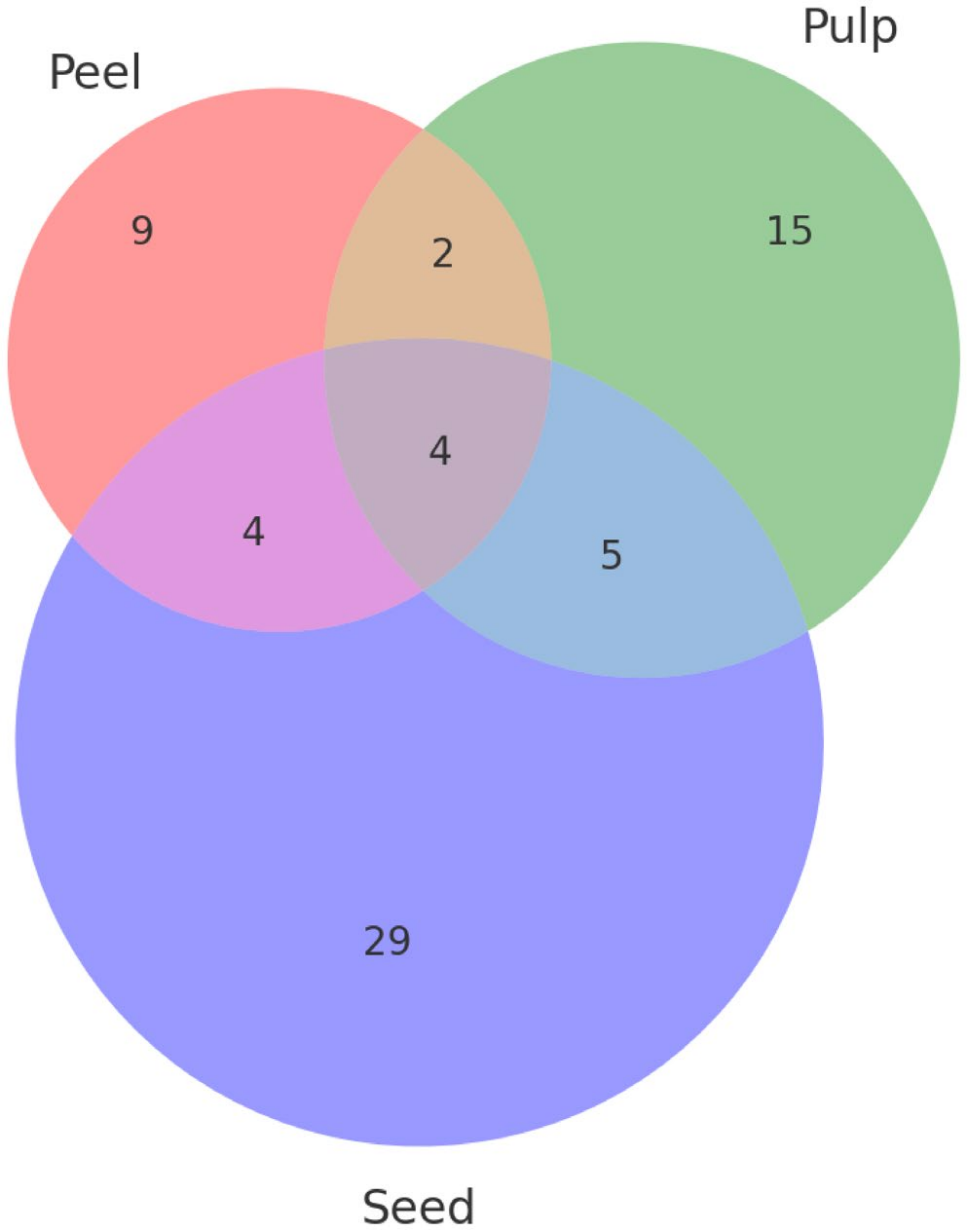
Veen's diagram of the amount of metabolites present in each part of this matrix.

For each metabolite, information was gathered regarding its PubChem ID, common name, HMDB code, adduct, signal‐to‐noise ratio (S/N), molecular formula, and its theoretically reported odor from the Flavornet database. Table 1 presents the complete list of identified metabolites, which constitute the volatilome of Hass avocado across the peel, pulp, and seed. In total, 19 compounds were identified in the peel, 26 in the pulp, and 42 in the seed, resulting in a total of 87 natural products forming the Hass avocado volatilome. A Venn diagram (Figure 2) illustrates the distribution of these metabolites: four were present in all three parts of the fruit, two were common to both peel and pulp, five were shared between pulp and seed, and four were found in both peel and seed (King‐Loeza et al. 2023). Additionally, nine metabolites were unique to the peel, 15 to the pulp, and 29 to the seed. This data highlights that the highest number of VOCs was detected in the seed, where terpenes were the predominant metabolite class.

**TABLE 1:** Volatile organic compounds reported theoretically and experimentally in peel, pulp and seed of Hass avocado.

Number of compounds identified	VOCs reported in the literature for Hass avocado	VOCs experimentally identified in peel			VOCs experimentally identified in pulp			VOCs experimentally identified in seed		
		Compound name	RT (min)	Exp KI	Compound name	RT (min)	Exp KI	Compound name	RT (min)	Exp KI
1	Hexanal (Lara‐García et al. 2021)	Acetone	2.004	—	Acetone	2.004	—	Acetone	2.004	—
2	(E)‐2‐Hexenal (Lara‐García et al. 2021)	Butanone	2.485	623.49	Pentanal	3.400	713	4‐methyl‐Cyclopentene	2.890	677.85
3	α‐Pinene (Vargas‐Abasolo et al. 2022)	Formic acid	5.180	782.21	Hexanal	5.424	792	Hexanal	5.424	791.64
4	Bicyclo[2.2.1]heptane, 2,2‐dimethyl‐3‐methylene (Vargas‐Abasolo et al. 2022)	Hexanal	5.424	791.64	(E)‐2‐Hexenal	7.370	855	α‐Pinene	10.335	937.23
5	β‐Pinene (Vargas‐Abasolo et al. 2022)	Furfural	6.850	838.6	Cyclohexanone	8.960	904	Camphene	11.125	956.09
6	1‐Hepten‐6‐one, 2‐methyl (Vargas‐Abasolo et al. 2022)	(E)‐2‐Hexenal	7.118	847.15	Nonanal	18.140	1111	(+)‐β‐Pinene	12.015	977.33
7	β‐Myrcene (Vargas‐Abasolo et al. 2022)	Cyclohexanone	8.960	904.42	(Z)‐2‐Tridecene	22.705	1211	β‐Myrcene	12.590	991.05
8	α‐Thujene (Vargas‐Abasolo et al. 2022)	β‐Myrcene	12.590	991.05	α‐Ylangene	26.940	1379	(+)‐2‐Carene	13.780	1017.5
9	Limonene (Vargas‐Abasolo et al. 2022)	(E)‐β‐ocimene	15.285	1049.7	(E)‐α‐Bergamotene	28.050	1443	(E)‐3‐Caren‐2‐ol	14.233	1027.2
10	(Z)‐β‐Ocimene (Vargas‐Abasolo et al. 2022)	Dodecanal	23.160	1226.1	Caryophyllene	28.335	1461	α‐Terpinolene	14.250	1027.5
11	(E)‐β‐Ocimene (Vargas‐Abasolo et al. 2022)	α‐Ylangene	26.940	1379.1	Undecylbenzene	31.730	1714	Limonene	14.425	1031.3
12	n‐Undecane (Vargas‐Abasolo et al. 2022)	(−)‐β‐Bourbonene	27.225	1393.3	1‐Pentadecene	31.820	1722	p‐Cymene	14.655	1036.2
13	Linalool (Vargas‐Abasolo et al. 2022)	2‐methylDecane	27.500	1408.8	7‐[(1E)‐1‐Propenyl]bicyclo[4.2.0]oct‐1‐ene	32.290	1763	(E)‐β‐ocimene	15.285	1049.7
14	Nonanal (Vargas‐Abasolo et al. 2022)	Caryophyllene	27.875	1432.2	2‐Isopropyl‐2,3,3a,4,7,7a‐hexahydro‐1H‐inden	33.920	1912	δ‐3‐Carene	15.748	1059.6
15	Hexanoic acid, butyl ester (Vargas‐Abasolo et al. 2022)	(Z,E)‐alpha‐Farnesene	28.086	1445.4	Dodecylbenzene	34.055	1926	γ‐terpinene	16.225	1069.8
16	n‐Dodecane (Vargas‐Abasolo et al. 2022)	(−)‐Germacrene D	28.770	1488.1	1‐(1‐Heptadecynyl)cyclopentanol	34.145	1934	Terpinolene	17.560	1098.3
17	Estragole (Vargas‐Abasolo et al. 2022)	(+)‐δ‐Cadinene	29.413	1532.9	1‐Nonylindane	34.420	1961	1,2,3,4‐tetramethylbenzene	19.175	1132.4
18	n‐Tridecane (Vargas‐Abasolo et al. 2022)	Epizonarene	29.510	1540	1‐Octyl‐1,2,3,4‐tetrahydronaphthalene	34.610	1980	1,2‐Dimethylindane	22.770	1212.9
19	α‐Cubebene (Lara‐García et al. 2021; Vargas‐Abasolo et al. 2022)	Undecylbenzene	31.730	1714	Palmitic acid	34.944	2013	10‐Undecenal	25.598	1312.1
20	Eugenol (Vargas‐Abasolo et al. 2022)				(E)‐hexadec‐9‐enoic acid	36.731	2202	α‐Ylangene	26.940	1379.0
21	Ylangene (Vargas‐Abasolo et al. 2022)				1‐3‐Dimethyladamantane	37.530	2291	(−)‐α‐Copaene	27.060	1385.0
22	α‐Copaene (Vargas‐Abasolo et al. 2022)				6‐Pentadecanone	37.770	2319	(−)‐Aromadendrene	27.155	1389.7
23	n‐Tetradecane (Vargas‐Abasolo et al. 2022)				γ‐Stearolactone	38.147	2363	Eremophilene	27.155	1389.7
24	Z‐α‐Bergamotene (Vargas‐Abasolo et al. 2022)				Heptadecane	38.720	2431	β‐Maaliene	28.555	1474.6
25	β‐Caryophyllene (Lara‐García et al. 2021; Vargas‐Abasolo et al. 2022)				2‐Methyloctacosane	40.916	2710	(Z)‐β‐Guaiene	28.715	1484.6
26	E‐α‐Bergamotene (Vargas‐Abasolo et al. 2022)				Docosane	41.165	2743	α‐Amorphene	28.775	1488.4
27	α‐Caryophyllene (Vargas‐Abasolo et al. 2022)							(+)‐δ‐amorphene	28.825	1491.5
28	Naphthalene, 1,2,3,4,4a,7‐hexahydro‐1,6‐dimethyl‐4‐(1‐methylethyl)‐ (Vargas‐Abasolo et al. 2022)							(E)‐Muurola‐4(14), 5‐diene	28.995	1502.5
29	β‐Copaene (Vargas‐Abasolo et al. 2022)							δ‐Selinene	29.070	1508
30	γ‐Elemene (Vargas‐Abasolo et al. 2022)							Epizonarene	29.180	1516
31	Tricyclo[4.4.0.02,7]dec‐3‐ene, 1,3‐dimethyl‐8‐(1‐methylethyl)‐,stereoisomer (Vargas‐Abasolo et al. 2022)							(+)‐δ‐Cadinene	29.413	1540
32	(Z)‐Calamenene (Vargas‐Abasolo et al. 2022)							Ω‐Cadinene	29.530	1541.4
33	α‐Farnesene (Vargas‐Abasolo et al. 2022)							(−)‐α‐Cubebene	29.675	1552
34	Acetone (Lara‐García et al. 2021; Vargas‐Abasolo et al. 2022)							Isocalamenene	29.705	1554.1
35	Acetaldehyde (Lara‐García et al. 2021)							Undecylbenzene	31.730	1713.9
36	Etanol (Lara‐García et al. 2021)							7‐[(1E)‐1‐Propenyl]bicyclo[4.2.0]oct‐1‐ene	32.290	1762.8
37	2‐Propanol (Lara‐García et al. 2021)							Dodecylbenzene	34.055	1925.4
38	2‐Methylpropanal (Lara‐García et al. 2021)							1‐(1‐Heptadecynyl)cyclopentanol	34.145	1934.3
39	2‐Methylpentane (Lara‐García et al. 2021)							1‐Nonylindane	34.420	1961.2
40	3‐Methylpentane (Lara‐García et al. 2021)							γ‐Stearolactone	38.147	2362.8
41	Propanol (Lara‐García et al. 2021)							Hexacosane	39.591	2538.4
42	Hexane (Lara‐García et al. 2021)							2‐methyloctacosane	40.916	2709.5
43	2‐Methylfuran (Lara‐García et al. 2021)									
44	Ethyl acetate (Lara‐García et al. 2021)									
45	2‐Methylpropanol (Lara‐García et al. 2021)									
46	3‐Methylbutanal (Lara‐García et al. 2021)									
47	Pentanal (Lara‐García et al. 2021)									
48	3‐Pentanone (Lara‐García et al. 2021)									
49	3‐Methylbutanol (Lara‐García et al. 2021)									
50	3‐Hidroxi‐butan‐2‐ ona (Lara‐García et al. 2021)									
51	2‐Metil butanol (Lara‐García et al. 2021)									
52	1‐Octeno (Lara‐García et al. 2021)									
53	Trans‐hex‐3‐enal (Lara‐García et al. 2021)									
54	Octano (Lara‐García et al. 2021)									
55	(Z)‐hex‐2‐enal (Lara‐García et al. 2021)									
56	(E)‐hex‐3‐en‐1‐ol (Lara‐García et al. 2021)									
57	(E)‐hex‐2‐en‐1‐ol (Lara‐García et al. 2021)									
58	Hexan‐1‐ol (Lara‐García et al. 2021)									
59	Heptanal (Lara‐García et al. 2021)									
60	Nonano (Lara‐García et al. 2021)									
61	2‐Nonanona (Lara‐García et al. 2021)									
62	2‐Metil‐5‐hepten‐ 2‐ona (Lara‐García et al. 2021)									
63	Octanal (Lara‐García et al. 2021)									
64	Trimetilbenceno (Lara‐García et al. 2021)									
65	Decanal (Lara‐García et al. 2021)									
66	α‐Copaene (Lara‐García et al. 2021; Vargas‐Abasolo et al. 2022)									
67	β‐Gurjunene (Lara‐García et al. 2021)									
68	δ‐Cadinene (Lara‐García et al. 2021)									
69	α‐Humulene (Lara‐García et al. 2021; Vargas‐Abasolo et al. 2022)									
70	Heptadecanal (Lara‐García et al. 2021)									
71	Tetradecanal (Lara‐García et al. 2021)									
72	Tetradecanoic acid (Lara‐García et al. 2021)									
73	Nonadecane (Lara‐García et al. 2021)									
74	Eicosane (Lara‐García et al. 2021)									
75	Alloaromadendrene (Vargas‐Abasolo et al. 2022)									
76	α‐Muurolene (Vargas‐Abasolo et al. 2022)									
77	γ‐Cadinene (Vargas‐Abasolo et al. 2022)									
78	α‐Calacorene (Vargas‐Abasolo et al. 2022)									

Abbreviations: KI exp, experimental kovats index; RT (min), experimental retention time.

A literature search was conducted to identify the main VOCs previously reported in Hass avocado, allowing for the confirmation of metabolites detected in this study while also revealing new terpenoid VOCs (Table 1). The identified metabolites were classified into three categories: known‐known, known‐unknown, and unknown‐unknown. The known‐known category includes compounds that have been previously reported and were identified in this study with high precision, such as hexanal, trans‐2‐hexenal, β‐myrcene, α‐pinene, β‐pinene, acetone, caryophyllene, cubebene, and copaene.

The known‐unknown category consists of compounds that were identified in this study but have not been previously reported in this matrix, including eremophilene, epizonarene, isocalamenene, maaliene, and (Z)‐β‐guaiene. Additionally, some analytical signals fell into the unknown‐unknown category, as they could not be identified with ≥ 90% similarity. These unidentified signals are expressed in terms of their retention time within the volatilomic profiles of the peel (Table S1), pulp (Table S2), and seed (Table S3), along with their peak area from GC–MS analysis. The metabolite diversity is marked by carbon–carbon double bonds, typical of alkenes and essential for terpenoid biosynthesis. These compounds exhibit a high degree of chemodiversity, often incorporating oxygenated functional groups such as aldehydes, ketones, alcohols and carboxylic acids. To explore the structural relationships between the detected metabolites, the Global Natural Products Social Molecular Networking (GNPS) (Martins et al. 2020), database was used to construct a molecular network graph, correlating the mass‐to‐charge (m/z) values obtained experimentally from the GC–MS analysis.

**FIGURE 3:**
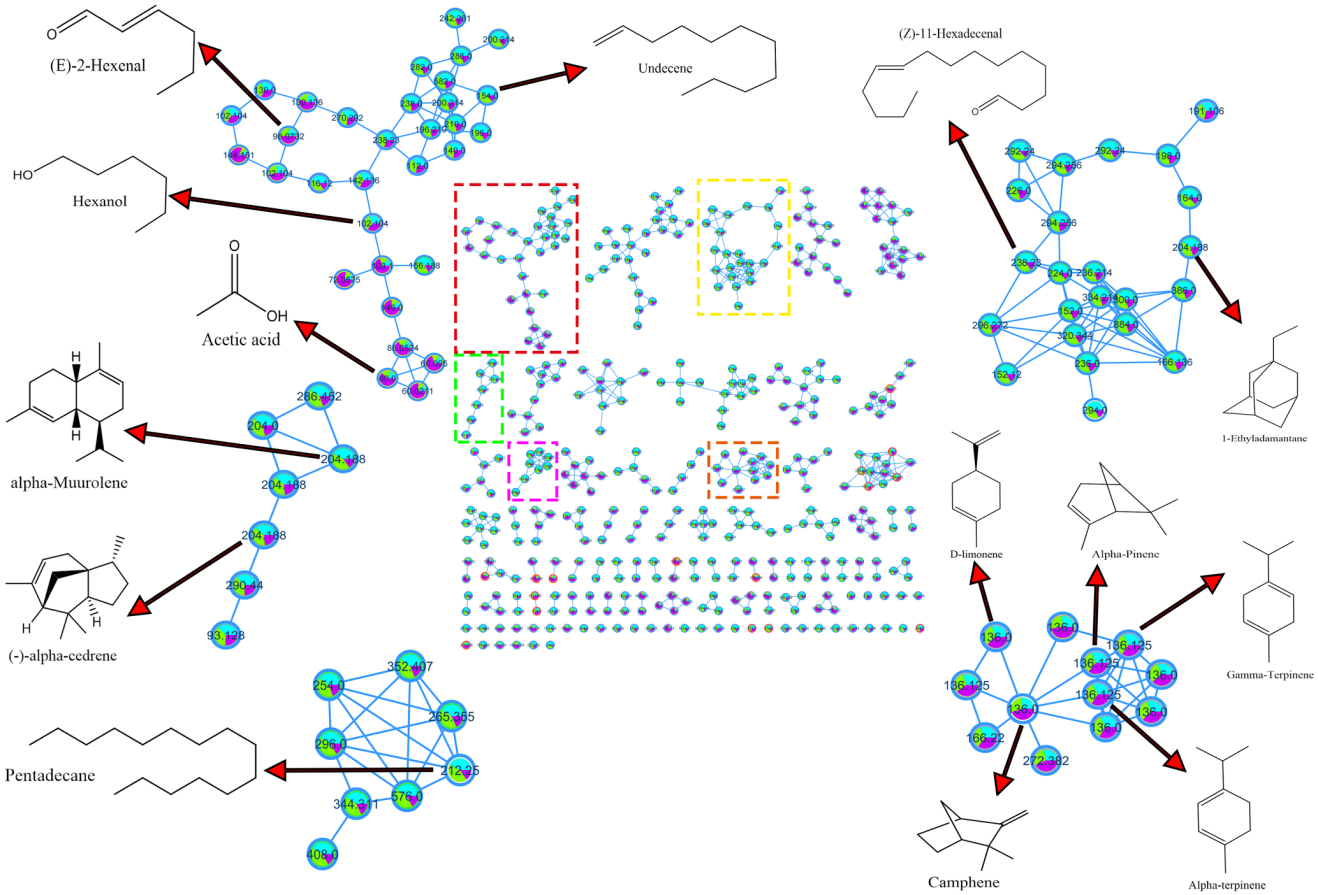
Analysis by family of compounds present in peel, pulp, and seed of Hass avocado. Group 1 red color: Compounds with alkenes, aldehydes, alcohols, and carboxylic acids. Group 2 green color: Sesquiterpenes type compounds. Group 3 pink color: Alkane‐type compounds. Group 4 yellow color: Bicyclic compounds and compounds with two or more functional groups. Group 5 orange color: Monoterpenes type compounds.

A molecular network was constructed using the GNPS (Global Natural Products Social Molecular Networking) platform based on MS fragmentation patterns of the detected VOCs. Spectral clustering was performed with a cosine similarity threshold of 0.7, a minimum of 6 matched fragment peaks, and a precursor ion mass tolerance of 2.0 Da. In the resulting network, nodes represent distinct m/z values, and their sizes correspond to the relative peak intensities obtained from GC–MS. Edges between nodes reflect spectral similarity, with thicker lines indicating higher cosine similarity scores. Figure 3 illustrates this molecular network, where compounds with structurally similar fragmentation patterns are grouped into five tentative chemical families, each highlighted with a colored box. These clusters include a variety of metabolite types: Group 1 (Red): Alkenes, aldehydes, alcohols, and carboxylic acids. Group 2 (Green): Sesquiterpenes. Group 3 (Pink): Alkanes. Group 4 (Yellow): Bicyclic compounds and multifunctional molecules. Group 5 (Orange): Monoterpenes.

Several compounds were tentatively annotated based on spectral matches, including (E)‐2‐Hexenal, Hexanol, Acetic Acid, α‐Muurolene, Undecene, α‐Pinene, Camphene, and others. Among these, Cluster 1, which includes D‐limonene, alpha‐pinene, and α‐terpinene, stands out for its strong contribution to sample differentiation, especially among avocado tissues. This classification underscores the chemical diversity present in the Hass avocado volatilome and supports the biological relevance of structurally distinct VOC families.

### 3.2 Theoretical Aroma Profile Based on VOC Identification

The theoretical aroma profile of Hass avocado was constructed by mapping each identified VOCs to its corresponding odor descriptor, as reported in the Flavornet database. This approach is based solely on the presence or absence of VOCs and does not include quantitative information such as peak areas, internal standards, or odor threshold values. Therefore, the resulting profile is exploratory in nature and should not be interpreted as a validated olfactometric analysis.

**FIGURE 4:**
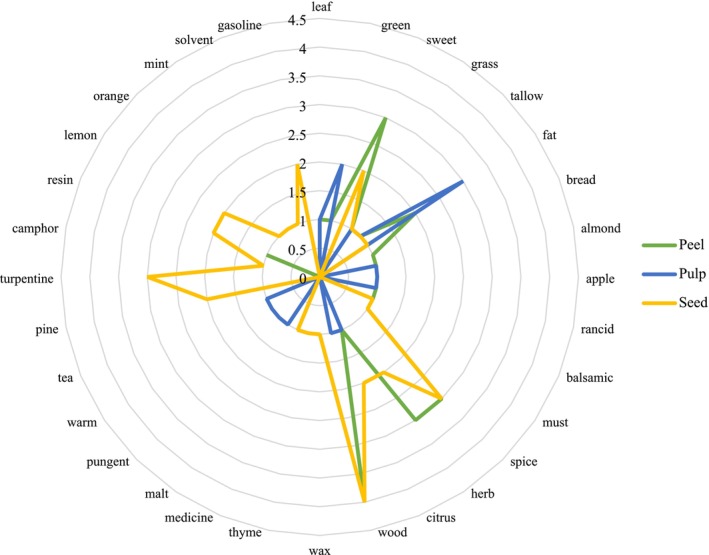
Olfactometric analysis for Hass avocado peel, pulp, and seed. Units: Olfactory intensity. Own elaboration.

Figure 4 presents a comparative overview of the theoretical aroma profiles for the peel (green), pulp (blue), and seed (yellow) tissues. The peel is predominantly associated with herbal, green, and citrus notes, likely reflecting a higher presence of terpenes and oxygenated compounds that contribute to fresh, aromatic characteristics. The pulp exhibits a stronger representation of fruity, almond‐like, and sweet descriptors, which may be attributed to esters and volatile aldehydes, alongside more pronounced fatty aromas. In contrast, the seed shows a clear dominance of resinous, woody, and medicinal notes, consistent with a greater abundance of sesquiterpenes and phenolic compounds, accompanied by mild sweet undertones.

This theoretical association between detected VOCs and their odor properties provides added value to the Hass avocado matrix by offering insight into its potential aromatic qualities. Such profiles may aid in the development of high‐quality export products and serve as a reference for sensory authenticity and product differentiation. The clear separation of volatile signatures between the three tissues also suggests distinct biological roles—such as defensive compounds in the seed and attractant volatiles in the pulp—possibly linked to ecological functions like herbivore deterrence or seed dispersal.

Nonetheless, a key limitation of this method is the absence of quantitative data and sensory confirmation. Without odor activity values (OAVs)—which relate compound concentration to human odor detection thresholds—or sensory panel evaluation, the actual impact of these compounds on perceived aroma cannot be determined. Future studies should incorporate OAV analysis or olfactory testing to prioritize impactful compounds and validate the sensory relevance of these findings in Hass avocado.

### 3.3 Multivariate Analysis

**FIGURE 5:**
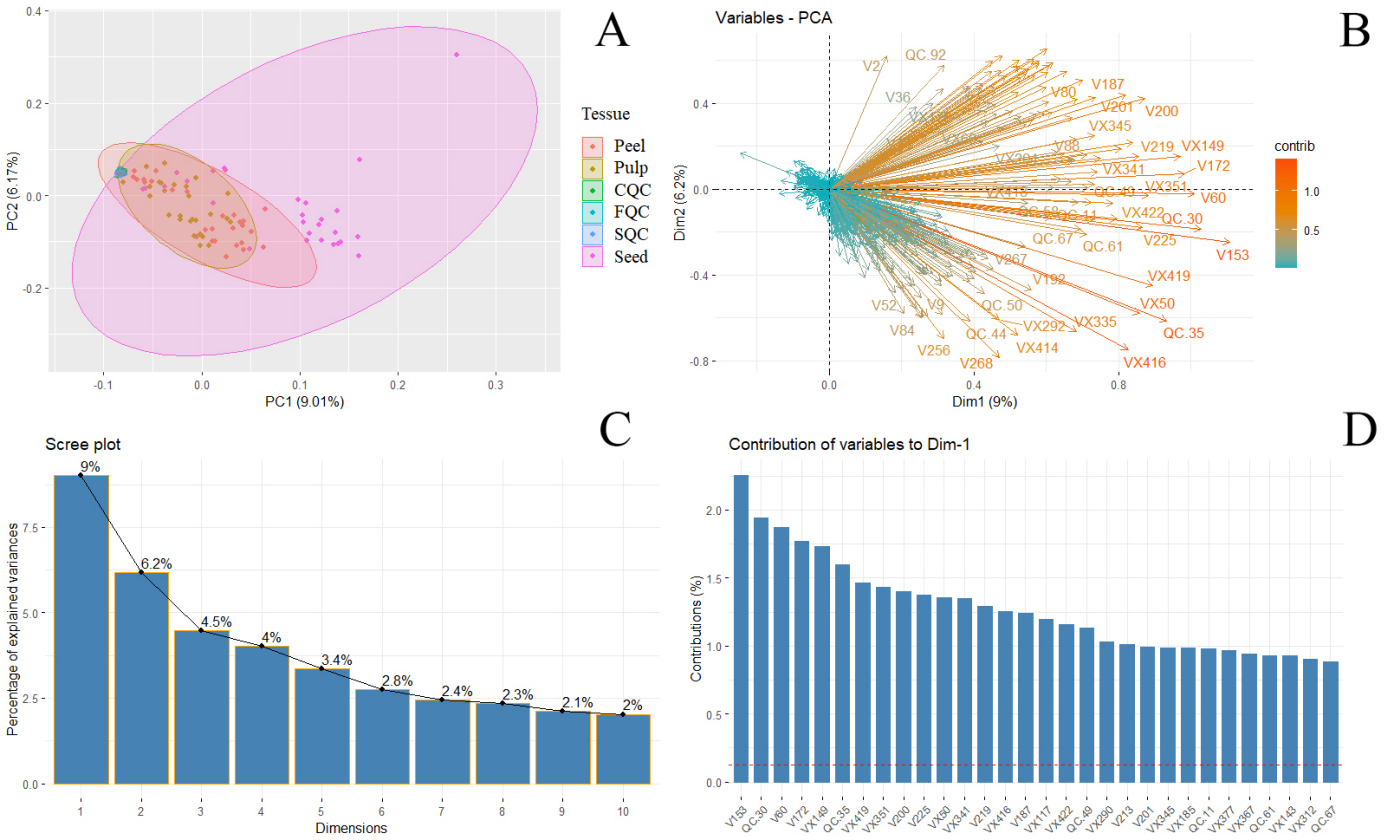
(A) Principal component analysis according to the contribution made by the total number of variables. (B) Principal component analysis according to the type of sample by working tissue. (C) Number of components for dimensionality reduction. (D) Contribution analysis on the variability of the data according to the metabolites.

A total of 33 peel samples, 33 pulp samples, and 33 seed samples were analyzed, allowing the identification of 702 variables, including known and unknown metabolites, which constitute the volatilome of the studied samples. Additionally, information related to 10 CQC, 12 SQC, and 13 FQC was incorporated. This multivariate dataset was processed using RStudio version 4.4.3. To assess multivariate normality, a Z‐score standardization was applied, revealing the need for non‐parametric statistical approaches for this dataset. To explore the data, a Principal Component Analysis (PCA) was performed to observe the distribution and behavior of the analyzed samples. The PCA results (Figure 5A) indicate that, as expected in biological data, there is a high degree of variability among samples. PC1 explains 9% of the variance, while PC2 accounts for 6.17%. This relatively low percentage is due to the fact that the PCA groups data from the peel, pulp, and seed in a single analysis, assessing the overall variability in the dataset. Notably, when PCA is performed separately for each fruit tissue, the percentage of variance explained by each principal component increases. However, since this study focuses on the volatile metabolites of Hass avocado as a whole, a global PCA analysis was prioritized.

#### 3.3.1 Main Contributors to Variability

The high variability in PC1 is primarily influenced by seed samples, which show the greatest variation, followed by pulp and peel. However, not all variables contribute equally to the differences between these tissues. Figure 5B presents a PCA loadings plot, highlighting the key variables responsible for the variability among peel, pulp, and seed samples. The variables were classified based on their identification codes: identified volatile metabolites (V) and unidentified volatile metabolites (VX). The most influential metabolites include V153, V60, V172, VX149, VX351, V200, V225, VX50, VX341, V219, VX416, V187, VX117, and VX422.

Figure 5C presents a scree plot, which suggests that the dataset lacks strong correlations among variables that would allow for higher variance explanation through fewer principal components. This indicates that more dimensions are required to effectively describe the dataset, reinforcing the need to perform dimensional PCA analyses to capture sample behavior more accurately. Figure 5D highlights the metabolites that contribute the most to the chemical diversity among peel, pulp, and seed samples. The most relevant compounds include V153 (bicyclogermacrene), V60 (β‐pinene), V172 (dodec‐(5Z)‐enyl acetate), V200 (cis‐β‐guaiene), V225 (α‐cadinene), V219 (δ‐cadinene), V187 (cis‐α‐bergamotene), V213 (amorpha‐4,7(11)‐diene), and V201 (α‐muurolene). These metabolites play a fundamental role in sample differentiation.

These metabolites drive the distribution of samples based on tissue type (peel, pulp, and seed), as observed in Figure 5A, where seed samples exhibit the highest variability, followed by pulp and peel. The reliability of this data exploration approach is supported by the clear clustering of quality control samples (CQC, SQC, FQC), forming a distinct point in multivariate space. This clustering confirms that the chromatographic system performed adequately, with no background noise interference, and that the analytical column remained in optimal condition throughout the injections. Furthermore, no memory effect or sample carryover was observed, validating the continuation of multivariate statistical analyses. Given these findings, a computational tool in Python is being developed to build classification models using bioinformatics‐based machine learning architectures.

#### 3.3.2 Environmental Influence on VOC Variability

These metabolites vary according to physicochemical factors that influence biochemical processes within the plant. Edaphoclimatic conditions and soil properties induce variations in the volatilomic profile, leading to the differential expression of certain metabolites depending on the geographical origin of the samples. Consequently, this type of sample analysis allows for classification based on the region of origin, since secondary metabolites present in the volatile profile are likely to vary under specific environmental conditions. To explore this phenomenon further, a Hierarchical Cluster Analysis (HCA) was conducted, applying a Euclidean distance metric to separate samples and determine their similarity levels, leading to the formation of distinct clustering groups.

#### 3.3.3 Regional Differentiation of Samples

**FIGURE 6:**
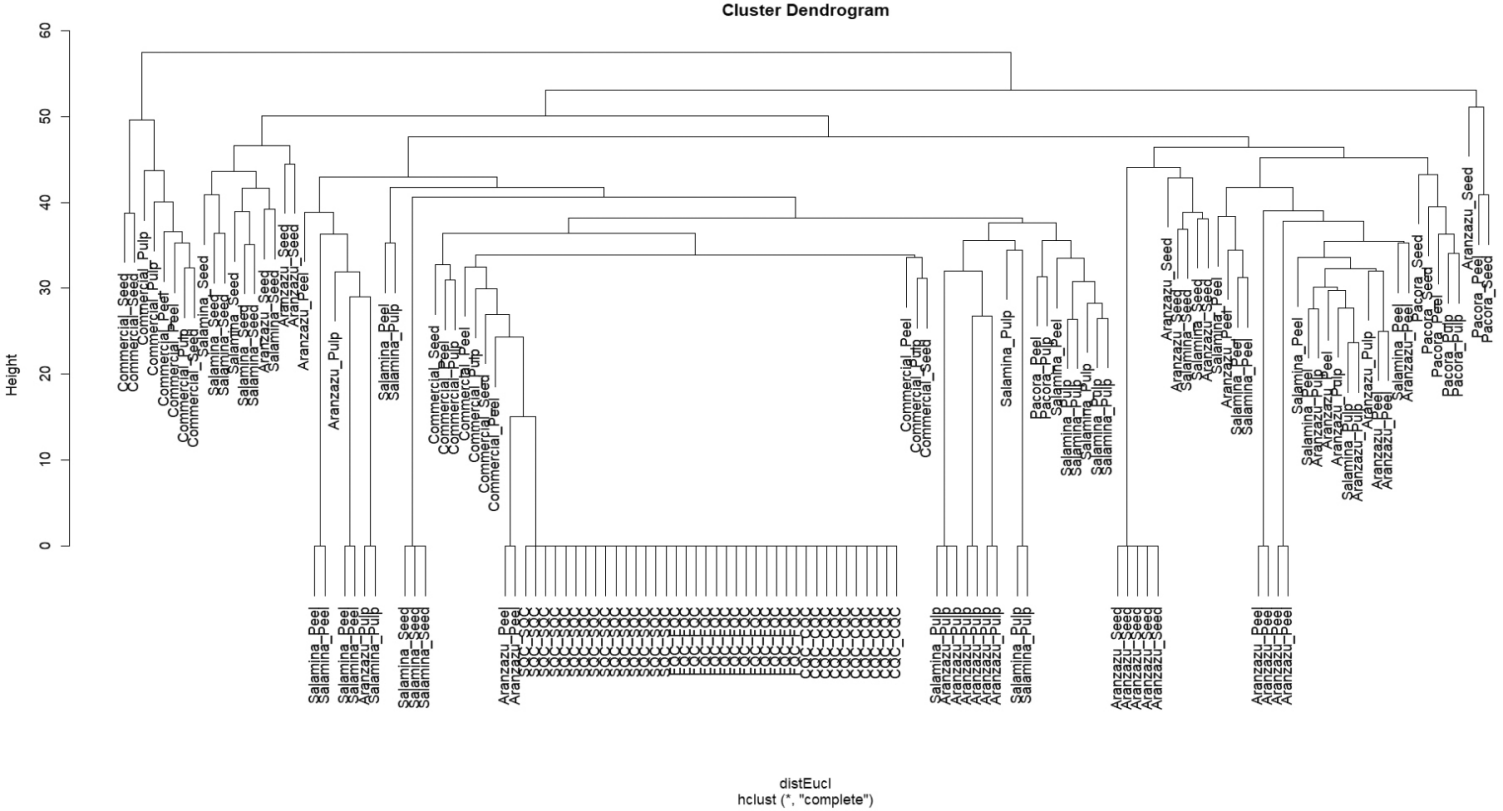
Hierarchical cluster analysis performed on the totality of the samples and the quality controls performed.

Figure 6 presents a hierarchical clustering analysis (HCA) of volatile compounds using Euclidean distance and complete linkage clustering, revealing distinct groupings among the analyzed samples. The first clustering group corresponds to quality control samples from the column and fiber, forming a well‐defined and separate cluster, confirming the reproducibility and stability of the instrumental analysis. Additionally, a clear differentiation is observed among peel, pulp, and seed samples from Aranzazu, Salamina, and Pacora, supporting the hypothesis that the volatile profiles vary significantly between these tissues and justifying their independent analysis. The samples from the three regions exhibit distinct clustering patterns, suggesting that edaphoclimatic conditions play a crucial role in shaping the volatilome composition of Hass avocado.

Notably, commercial samples tend to group closely with those from Pacora, highlighting a strong similarity in their volatile profiles. These results demonstrate that multivariate classification techniques can effectively differentiate samples based on their volatile composition, supporting their potential use in tracing geographic origin and quality assessment of Hass avocado.

### 3.4 Biochemical Pathways

To interpret the volatile metabolites detected in the peel, pulp, and seed of Hass avocado, a metabolic enrichment analysis was conducted using the hypergeometric test and relative topological centrality (betweenness centrality). The complete metabolome available in the selected pathway library served as a reference. The results revealed significant enrichment in several biochemical pathways within the volatilome profile. Notably, the most enriched pathways included sesquiterpenoid and triterpenoid biosynthesis (*p* = 9.05 × 10^−4^, −log(*p*) = 3.0432, impact = 0.0), pyruvate metabolism (*p* = 0.0034, −log(*p*) = 2.4684, impact = 0.0341), glycolysis and gluconeogenesis (*p* = 0.0043, −log(*p*) = 2.362, impact = 0.0316), fatty acid biosynthesis (*p* = 0.0195, −log(*p*) = 1.7104, impact = 0.0112), and cutin, suberin, and wax biosynthesis (*p* = 0.0638, −log(*p*) = 1.195, impact = 0.0). Other identified pathways included biosynthesis of unsaturated fatty acids, fatty acid elongation, glycosylphosphatidylinositol (GPI)‐anchor biosynthesis, and fatty acid degradation, all of which contribute to the complex metabolic landscape of the fruit's volatilome.

**FIGURE 7:**
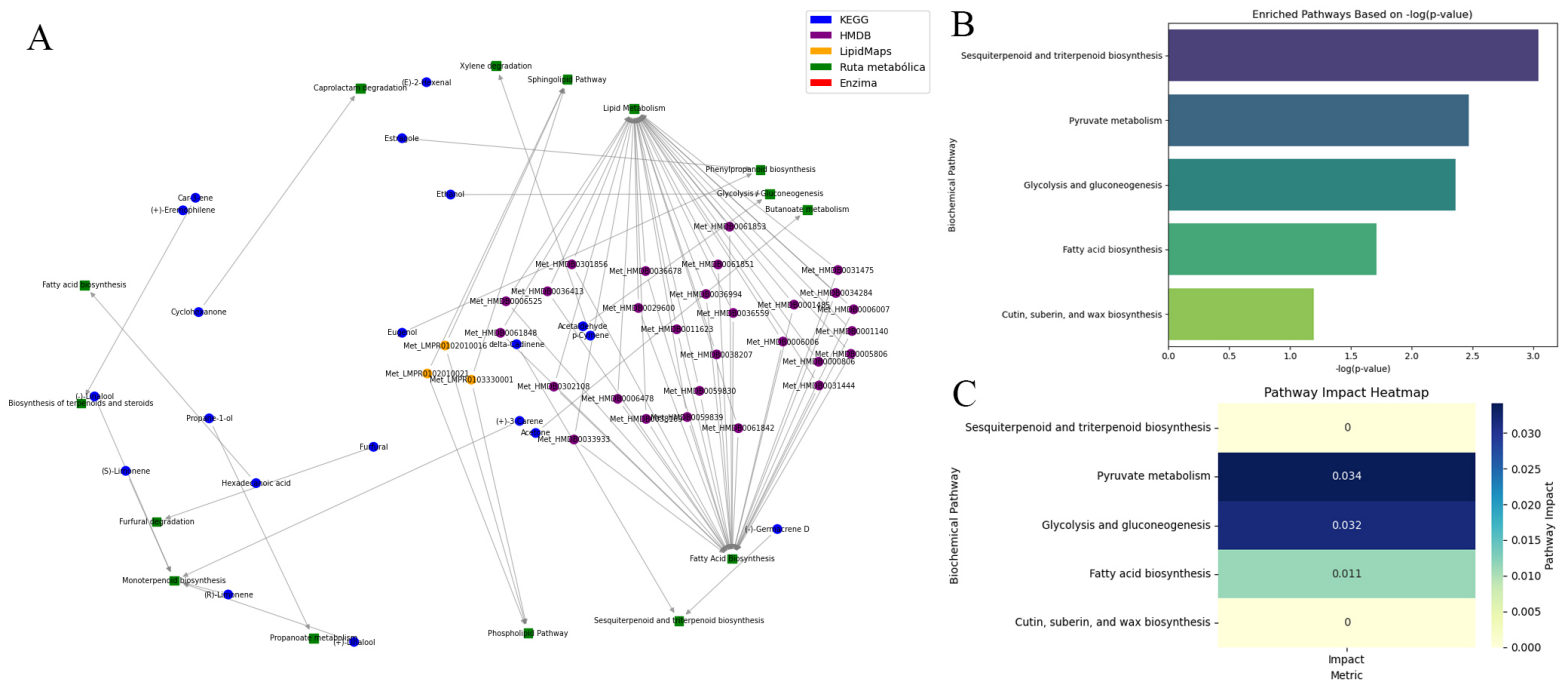
The biochemical pathways involved in the formation of the different natural products identified by GC–MS are listed in Table 1. (A) Metabolite‐pathway interaction network. (B) Bar graph of pathway enrichment. (C) Heat map of the topological impact of the pathways.

Additionally, using a Python‐based algorithm to search for KEGG pathway codes associated with detected metabolites (Figure 7A), other relevant biochemical pathways were identified, including furfural degradation, caprolactam degradation, biosynthesis of terpenoids and steroids, phenylpropanoid biosynthesis, monoterpenoid biosynthesis, xylene degradation, propanoate metabolism, and butanoate metabolism. The identified pathways highlight the metabolic diversity of volatile compounds derived from various biochemical processes. The presence of pathways related to terpenoid, sesquiterpenoid, and monoterpenoid biosynthesis suggests that these compounds play a fundamental role in the aromatic profile of Hass avocado. Furthermore, the identification of fatty acid metabolism pathways indicates a significant contribution of lipophilic compounds to the volatilome. The detection of furfural, caprolactam, and xylene degradation pathways suggests possible metabolic transformation mechanisms that lead to characteristic volatile compounds. The involvement of glycolysis, gluconeogenesis, and pyruvate metabolism establishes a connection between primary energy metabolism and the production of secondary volatile metabolites. This underscores the biochemical complexity of the Hass avocado volatilome and lays the foundation for future studies on environmental factors influencing volatile compound composition.

#### 3.4.1 Key Metabolic Pathways and Their Roles in the Volatilome

##### 3.4.1.1 Sesquiterpenoid and Triterpenoid Biosynthesis

Sesquiterpenoids (C_15_) and triterpenoids (C_30_) are synthesized through two main biochemical pathways: the mevalonate (MVA) pathway in the cytoplasm and the methylerythritol phosphate (MEP) pathway in plastids. Sesquiterpenoids originate from the cyclization of farnesyl pyrophosphate (FPP), while triterpenoids are derived from squalene. These classes of compounds play vital roles in plant defense mechanisms and contribute significantly to the aroma profile of essential oils (Dudareva et al. 2013).

Figure 7B displays a bar chart representing the statistical significance of each enriched pathway, calculated using a hypergeometric test. The *y*‐axis lists the identified biochemical pathways, while the *x*‐axis shows the −log(*p*‐value). A higher −log(*p*‐value) indicates stronger statistical enrichment, meaning that the presence of associated metabolites is unlikely to occur by chance. In this context, the sesquiterpenoid and triterpenoid biosynthesis pathway exhibited the highest −log(*p*‐value = 3.0432), indicating significant overrepresentation among the detected volatile metabolites.

However, Figure 7C presents a heatmap of pathway impact, based on betweenness centrality within the biochemical network. This metric reflects how central each detected metabolite is within its respective pathway—metabolites located at key branching points or hubs receive higher scores. Interestingly, while the sesquiterpenoid and triterpenoid biosynthesis pathway demonstrated strong statistical enrichment, its pathway impact score was zero. This suggests that although multiple metabolites from this pathway were detected, they occupy peripheral positions within the network, rather than central, high‐impact nodes involved in key metabolic steps.

##### 3.4.1.2 Pyruvate Metabolism and Glycolysis/Gluconeogenesis

Pyruvate metabolism links glycolysis to the Krebs cycle and acts as a precursor for various volatile compounds (Kanehisa et al. 2020). Pyruvate is converted into Acetyl‐CoA, a key intermediate in lipid and terpenoid biosynthesis. This pathway is vital for energy production and the synthesis of both primary and secondary metabolites (Sweetlove et al. 2010).

##### 3.4.1.3 Fatty Acid Biosynthesis and Lipid Metabolism

Fatty acid biosynthesis primarily occurs in plastids, where Acetyl‐CoA is carboxylated to malonyl‐CoA, which is then elongated into long‐chain fatty acids. The endoplasmic reticulum (ER) further elongates these fatty acids, forming waxes and structural lipids that influence fruit quality and volatile precursor formation (Fatiha 2020).

##### 3.4.1.4 Phenylpropanoid and Minor Terpenoid Biosynthesis

Phenylpropanoids originate from phenylalanine conversion into cinnamic acid, leading to the formation of volatile phenolic compounds. Monoterpenoids (C_10_), derived from the MEP pathway, contribute to the aroma of fruits and essential oils, influencing fruit–insect interactions (Qiao et al. 2021).

##### 3.4.1.5 Butanoate Metabolism and Volatile Ester Formation

Butanoate metabolism plays a critical role in the synthesis of volatile esters, compounds responsible for fruity and sweet aromas in ripening fruits (El Hadi et al. 2013). These metabolic pathways are key in shaping sensory profiles and distinguishing fruit samples based on geographic origin (El Hadi et al. 2013).

#### 3.4.2 Biological Relevance of Enriched Pathways in Hass Avocado

The enrichment of the sesquiterpenoid and triterpenoid biosynthesis pathway aligns with prior findings on avocado's volatile profile. Compounds such as α‐caryophyllene, α‐muurolene, β‐pinene, and cis‐β‐guaiene—detected in this study—contribute to the distinctive aroma of Hass avocado and have been linked to antifungal, antibacterial, and anti‐inflammatory properties. Acting as phytoalexins, these terpenoids also enhance the fruit's natural defense against pests and pathogens. The enrichment of fatty acid biosynthesis and fatty acid degradation pathways is biologically expected, given that avocado pulp is rich in unsaturated fatty acids, including oleic, linoleic, and palmitoleic acid (Carvalho and Velásquez 2015). These lipids serve as precursors for a variety of aldehydes, esters, and ketones, which contribute to the fruit's fruity and fatty aroma profile (Yahia et al. 2025).

Additionally, the detection of glycolysis and pyruvate metabolism pathways highlights their role in energy production and the generation of precursors for volatile compounds such as acetic acid, ethanol, and acetaldehyde—metabolites previously linked to avocado ripening and softening (Pesis 2005). Overall, the biochemical pathways identified through enrichment analysis not only align with the volatilomic data generated in this study but also corroborate previous findings on avocado metabolism, supporting the robustness and biological significance of the results.

## 4 Conclusions

The peel, pulp, and seed of Hass avocado exhibit unique volatile profiles, with terpenes dominating the seed, esters and aldehydes in the pulp, and oxygenated terpenes in the peel. These variations suggest tissue‐specific metabolic roles, such as seed defense (woody, medicinal notes) and pulp‐mediated dispersal (fruity, sweet notes). The enrichment analysis identified sesquiterpenoid and triterpenoid biosynthesis, fatty acid metabolism, glycolysis, and pyruvate metabolism as key contributors to volatile production. These pathways provide precursors for terpenes, aldehydes, and esters, influencing aroma, fruit quality, and plant defense mechanisms.

PCA and HCA analyses confirmed high variability in the seed volatilome, followed by the pulp and skin. Pooling of quality control samples (CQC, SQC, FQC) validated the reliability of the GC–MS data, ensuring that the differences were biological and not instrumental. The highest VOC concentration was detected in the middle of the fruit, however it is important to follow student the distribution of metabolites along the fruit surface, which highlights the variation of metabolic flux in the fruit. This suggests that biosynthetic activity is spatially regulated, influencing the composition of volatiles at different locations in the fruit. The combination of GC–MS, metabolic enrichment analysis, and a Python‐based KEGG search algorithm allowed a comprehensive identification of pathways and metabolite interactions, offering a robust analytical approach for future volatilomics studies.

As a future perspective, from the development of this research, a development model based on a machine learning model has been built for the predictive classification of avocado samples based on volatile composition and the expression of external contaminants. These results highlight the biochemical complexity of Hass avocado and its potential applications in sensory analysis, industrial use and metabolic research.

## Author Contributions

**Juan Pablo Betancourt Arango:** methodology (equal), writing – original draft (equal). **Alejandro Patiño Ospina:** investigation (equal), software (equal). **Jhon Alexander Fiscal Ladino:** formal analysis (equal), visualization (equal). **Gonzalo Taborda Ocampo:** supervision (equal), writing – review and editing (equal).

## Conflicts of Interest

The authors declare no conflicts of interest.

## Supporting information


**Table S1.** Volatile organic compounds obtained experimentally in Hass avocado peel.
**Table S2.** Volatile organic compounds experimentally obtained in Hass avocado pulp.
**Table S3.** Volatile organic compounds experimentally obtained in Hass avocado seed.

## Data Availability

The data supporting the findings of this study are included in the Supporting Information files provided with the manuscript.
