# Supplementary material for: Volatilomic Analysis in Peel, Pulp and Seed of Hass Avocado ( Persea americana Mill.) From the Northern Subregion of Caldas by Gas Chromatography With Mass Spectrometry
Source: Food Sci Nutr. 2025 Jul 7;13(7):e70489. doi: 10.1002/fsn3.70489 (PMC12231068; doi:10.1002/fsn3.70489)
Supplement: Supplementary file 1 — Table S1. Volatile organic compounds obtained experimentally in Hass avocado peel. Table S2. Volatile organic compounds experimentally obtained in Hass avocado pulp. Table S3. Volatile organic compounds experimentally obtained in Hass avocado seed. [file FSN3-13-e70489-s001.docx]

**Supplementary Material**

**Table S1.** Volatile organic compounds obtained experimentally in Hass avocado peel.

| # | Compound | Common name | Formula | m/z | RT | Exact Mass | BP (°C) | Exp KI | Theo KI | SI | Average area | %Error KI | Pubchem CID | | HMDB | Adduct | S/N | Odor |
| --- | --- | --- | --- | --- | --- | --- | --- | --- | --- | --- | --- | --- | --- | --- | --- | --- | --- | --- |
| 1 | Acetone | Acetone | C_3_H_6_O | 43 | 2.004 | 58.0418 | 56.08 | - | **-** | 97 | 1783361.28 | - | | 180 | HMDB0001659 | [M+H] | 18.45 | - |
| 2 | RT2.015 | - | - | 43 | 2.015 | - | - | - | - | - | 52233.4285 |  | | - | - | [M+H] | 21.93 | - |
| 3 | RT2.085 | - | - | 43 | 2.085 | - | - | - | - | - | 965488.714 | - | | - | - | [M+H] | 15.93 | - |
| 4 | RT2.110 | - | - | 203 | 2.110 | - | - | - | - | - | 414583.714 | - | | - | - | [M+H] | 1.26 | - |
| 5 | RT2.325 | - | - | 79 | 2.325 | - | - | 602.01 | - | - | 790091.714 | - | | - | - | [M+H] | 58.14 | - |
| 6 | Butanone | Butanone | C_4_H_8_O | 43 | 2.485 | 72.0575 | 79.59 | 623.49 | 582 | 92 | 1166408.42 | 7.2025332 | | 6569 | HMDB0000474 | [M+H] | 13.1 | - |
| 7 | RT2.872 | - | - | 56 | 2.872 | - | - | 675.44 | - | - | 101646.714 | - | | - | - | [M+H] | 4.06 | - |
| 8 | RT3.000 | - | - | 43 | 3.000 | - | - | 692.62 | - | - | 874107.429 | - | | - | - | [M+H] | 2.27 | - |
| 9 | RT3.070 | - | - | 147 | 3.070 | - | - | 700.58 | - | - | 2554.28571 | - | | - | - | [M+H] | 10.98 | - |
| 10 | RT3.150 | - | - | 147 | 3.150 | - | - | 703.68 | - | - | 447364 | - | | - | - | [M+H] | 18.95 | - |
| 11 | RT3.890 | - | - | 46 | 3.890 | - | - | 732.3 | - | - | 11065.8571 | - | | - | - | [M+H] | 12.77 | - |
| 12 | RT4.435 | - | - | 43 | 4.435 | - | - | 753.38 | - |  | 12601.1429 | - | | - | - | [M+H] | 1.55 | - |
| 13 | RT4.810 | - | - | 77 | 4.810 | - | - | 767.89 | - | - | 544378 | - | | - | - | [M+H] | 0.39 | - |
| 14 | Formic acid | Formic acid | CH_2_O_2_ | 46 | 5.180 | 46.0054 | 101 | 782.21 | 543 | 93 | 128327 | 44.052492* | | 284 | HMDB00142 | [M+H] | 76.18 | - |
| 15 | Hexanal | Hexanal | C_6_H_12_O | 43 | 5.424 | 100.088 | 129.6 | 791.64 | 769 | 99 | 94426.2857 | 2.9446165 | | 6184 | HMDB05994 | [M+H] | 97.44 | grass, tallow, fat |
| 16 | RT5.545 | - | - | 55 | 5.545 | - | - | 796.32 | - | - | 66856.7143 | - | | - | - | [M+H] | 7.14 | - |
| 17 | RT5.675 | - | - | 41 | 5.675 | - | - | 801.12 | - | - | 130363.714 | - | | - | - | [M+H] | 8.02 | - |
| 18 | RT5.730 | - | - | 43 | 5.730 | - | - | 802.87 | - | - | 17945.4286 | - | | - | - | [M+H] | 2.52 | - |
| 19 | Furfural | Furfural | C_5_H_4_O_2_ | 41 | 6.850 | 96.0211 | 161.7 | 838.6 | 847 | 91 | 207065.714 | 0.9570697 | | 7362 | HMDB32914 | [M+H] | 16.44 | bread, almond, sweet |
| 20 | \|trans\|-2-Hexenal | trans-2-Hexenal | C_6_H_10_O | 41 | 7.118 | 98.0731 | 47 | 847.15 | 820 |  | 849264.286 | 3.3103824 | | 5281168 | HMDB31496 | [M+H] | 958.93 | apple, green, leaf, fat, rancid |
| 21 | RT7.630 | - | - | 73 | 7.630 | - | - | 863.48 | - | - | 80091.7143 | - | | - | - | [M+H] | 91.05 | - |
| 22 | Cyclohexanone | Cyclohexanone | C_6_H_10_O | 55 | 8.960 | 98.0731 | 155.4 | 904.42 | 852 | 93 | 166118.714 | 6.1894182 | | 7967 | HMDB0003315 | [M+H] | 16.12 | - |
| 23 | RT9.140 | - | - | 281 | 9.140 | - | - | 908.71 | - | - | 126500 | - | | - | - | [M+H] | 10.05 | - |
| 24 | RT11.270 | - | - | 41 | 11.270 | - | - | 959.55 | - | - | 9108.14286 | - | | - | - | [M+H] | 8.83 | - |
| 25 | RT11.880 | - | - | 281 | 11.880 | - | - | 974.11 | - | - | 72659.8571 | - | | - | - | [M+H] | 0.79 | - |
| 26 | RT12.000 | - | - | 41 | 12.000 | - | - | 976.97 | - | - | 235907.429 | - | | - | - | [M+H] | 9.77 | - |
| 27 | RT12.105 | - | - | 110 | 12.105 | - | - | 979.47 | - | - | 53796.8571 | - | | - | - | [M+H] | 5.86 | - |
| 28 | RT12.150 | - | - | 55 | 12.150 | - | - | 980.55 | - |  | 185252 | - | | - | - | [M+H] | 7.83 | - |
| 29 | Myrcene | beta-Myrcene | C_10_H_16_ | 41 | 12.590 | 136.125 | 167 | 991.05 | 979 | 98 | 160872.286 | 1.23086 | | 31253 | HMDB0038169 | [M+H] | 299.2 | balsamic, must, spice |
| 30 | RT13.340 | - | - | 93 | 13.340 | - | - | 1008 | - | - | 36469 | - | | - | - | [M+H] | 94.08 | - |
| 31 | RT14.040 | - | - | 91 | 14.040 | - | - | 1023 | - | - | 5394.28571 | - | | - | - | [M+H] | 7.11 | - |
| 32 | RT14.220 | - | - | 119 | 14.220 | - | - | 1026.9 | - | - | 15582.2857 | - | | - | - | [M+H] | 46.54 | - |
| 33 | RT14.430 | - | - | 67 | 14.430 | - | - | 1031.4 | - | - | 9874.14286 | - | | - | - | [M+H] | 19.41 | - |
| 34 | RT14.605 | - | - | 43 | 14.605 | - | - | 1035.1 | - | - | 35245.4286 | - | | - | - | [M+H] | 61.87 | - |
| 35 | \|E\|-_b_-Ocimene | (E)-beta-ocimene | C_10_H_16_ | 93 | 15.285 | 136.125 | 174 | 1049.7 | 1041 | 100 | 120095.714 | 0.8336984 | | 5281553 | HMDB0030089 | [M+H] | 2.67 | sweet, herb |
| 36 | RT15.650 | - | - | 73 | 15.650 | - | - | 1057.5 | - |  | 8875.28571 | - | | - | - | [M+H] | 71.08 | - |
| 37 | RT15.810 | - | - | 93 | 15.810 | - | - | 1060.9 | - | - | 2923 | - | | - | - | [M+H] | 0.09 | - |
| 38 | RT16.000 | - | - | 73 | 16.000 | - | - | 1065 | - | - | 4914 | - | | - | - | [M+H] | 1.72 | - |
| 39 | RT17.085 | - | - | 43 | 17.085 | - | - | 1088.2 | - | - | 102057.714 | - | | - | - | [M+H] | 7.79 | - |
| 40 | RT17.110 | - | - | 41 | 17.110 | - | - | 1088.8 | - | - | 4855.57143 | - | | - | - | [M+H] | 5.19 | - |
| 41 | RT17.465 | - | - | 73 | 17.465 | - | - | 1096.4 | - | - | 55736.7143 | - | | - | - | [M+H] | 0.75 | - |
| 42 | RT18.635 | - | - | 41 | 18.635 | - | - | 1121.1 | - | - | 10899 | - | | - | - | [M+H] | 4.03 | - |
| 43 | RT20.205 | - | - | 73 | 20.205 | - | - | 1154.1 | - | - | 2608.42857 | - | | - | - | [M+H] | 2.63 | - |
| 44 | RT20.510 | - | - | 117 | 20.510 | - | - | 1160.5 | - | - | 10613.2857 | - | | - | - | [M+H] | 3.86 | - |
| 45 | RT21.940 | - | - | 207 | 21.940 | - | - | 1190.6 | - | - | 2703.85714 | - | | - | - | [M+H] | 0.67 | - |
| 46 | RT22.365 | - | - | 265 | 22.365 | - | - | 1199.6 | - | - | 17145.4286 | - | | - | - | [M+H] | 3.25 | - |
| 47 | RT22.480 | - | - | 43 | 22.480 | - | - | 1203.2 | - | - | 36631.2857 | - | | - | - | [M+H] | 8.62 | - |
| 48 | RT22.675 | - | - | 41 | 22.675 | - | - | 1209.8 | - | - | 67512.5714 | - | | - | - | [M+H] | 2.9 | - |
| 49 | Dodecanal | Dodecanal | C_12_H_24_O | 41 | 23.160 | 184.182 | 185 | 1226.1 | 1386 | 97 | 49339 | 11.53721 | | 8194 | HMDB0033933 | [M+H] | 11.06 | - |
| 50 | RT23.270 | - | - | 43 | 23.270 | - | - | 1229.8 | - | - | 9578.71429 | - | | - | - | [M+H] | 0.54 | - |
| 51 | RT23.455 | - | - | 55 | 23.455 | - | - | 1236 | - | - | 10791.4286 | - | | - | - | [M+H] | 2.1 | - |
| 52 | RT23.485 | - | - | 73 | 23.485 | - | - | 1237 | - | - | 4072 | - | | - | - | [M+H] | 26.77 | - |
| 53 | RT25.500 | - | - | 73 | 25.500 | - | - | 1307.2 | - | - | 308895.714 | - | | - | - | [M+H] | 99.01 | - |
| 54 | ,alpha,-ylangene | alpha-Ylangene | C_15_H_24_ | 105 | 26.940 | 204.187 | - | 1379.1 | - | - | 50197.7143 | - | | 442409 | - | [M+H] | 27.87 | - |
| 55 | Bourbonene <beta-> | (-)-beta-Bourbonene | C_15_H_24_ | 81 | 27.225 | 204.187 | 121 | 1393.3 | 1374 |  | 62614.7143 | 1.402244 | | 62566 | HMDB0038155 | [M+H] | 95.46 | herb |
| 56 | RT27.340 | - | - | 67 | 27.340 | - | - | 1399 | - | - | 36217.7143 | - | | - | - | [M+H] | 5.96 | - |
| 57 | Decane, 2-methyl- | 2-methyldecane | C_11_H_24_ | 43 | 27.500 | 156.187 | 189.3 | 1408.8 | 1066 |  | 13814.4286 | 32.165306* | | 23415 | HMDB0039000 | [M+H] | 16.22 | - |
| 58 | RT27.615 | - | - | 41 | 27.615 | - | - | 1415.9 | - | - | 17345.7143 | - | | - | - | [M+H] | 4.73 | - |
| 59 | RT27.810 | - | - | 55 | 27.810 | - | - | 1428.1 | - | - | 18115.5714 | - | | - | - | [M+H] | 4.1 | - |
| 60 | Bicyclo[7.2.0]undec-4-ene, 4,11,11-trimethyl-8-methylene-,[ | Caryophylene | C_15_H_24_ | 41 | 27.875 | 204.187 | 256 | 1432.2 | 1424 | 99 | 161810.143 | 0.5749649 | | 5281515 | HMDB0036792 | [M+H] | 19.82 | wood, spice |
| 61 | RT28.005 | - | - | 144 | 28.005 | - | - | 1440.3 | - | - | 8188.71429 | - | | - | - | [M+H] | 0.92 | - |
| 62 | 1,3,6,10-Dodecatetraene, 3,7,11-trimethyl-, (Z,E)- | (Z,E)-alpha-Farnesene | C_15_H_24_ | 41 | 28.086 | 204.187 | 279 | 1445.4 | 1486 | 98 | 27122.8571 | 2.7338493 | | 5362889 | HMDB0036066 | [M+H] | 3.14 | wood, sweet |
| 63 | Germacrene D | (-)-Germacrene D | C_15_H_24_ | 79 | 28.770 | 204.187 | - | 1488.1 | 1490 | 94 | 126153.429 | 0.1258389 | | 5317570 | HMDB0302240 | [M+H] | 4.92 | wood, spice |
| 64 | Cadinene <delta-> | (+)-delta-Cadinene | C_15_H_24_ | 119 | 29.413 | 204.187 | 121 | 1532.9 | - | 98 | 365617.143 | - | | 441005 | HMDB0035084 | [M+H] | 9.47 | thyme, medicine, wood |
| 65 | Epizonarene | Epizonarene | C_15_H_24_ | 81 | 29.510 | 204.187 | - | 1540 | 1499 | 92 | 49960 | 2.7351568 | | 595385 | - | [M+H] | 3.05 | - |
| 66 | RT29.715 | - | - | 159 | 29.715 | - | - | 1554.9 | - | - | 11515.8571 | - | | - | - | [M+H] | 28.09 | - |
| 67 | RT30.000 | - | - | 93 | 30.000 | - | - | 1575.6 | - | - | 8982.57143 | - | | - | - | [M+H] | 0.8 | - |
| 68 | RT30.165 | - | - | 67 | 30.165 | - | - | 1587.6 | - | - | 15766.2857 | - | | - | - | [M+H] | 5.81 | - |
| 69 | RT30.380 | - | - | 41 | 30.380 | - | - | 1603.6 | - | - | 44205.4286 | - | | - | - | [M+H] | 7.72 | - |
| 70 | RT30.580 | - | - | 71 | 30.580 | - | - | 1619.8 | - | - | 129964.571 | - | | - | - | [M+H] | 34.15 | - |
| 71 | Benzene, undecyl- | Undecylbenzene | C_17_H_28_ | 92 | 31.730 | 232.219 | 316 | 1714 | 1760 | 90 | 405850.143 | 2.6217645 | | 23194 | - | [M+H] | 94.18 | - |
| 72 | RT34.580 | - | - | 149 | 34.580 | - | - | 1977 | - | - | 2851.14286 | - | | - | - | [M+H] | 21.58 | - |
| 73 | RT34.974 | - | - | 43 | 34.974 | - | - | 2016.3 | - | - | 28305885.6 | - | | - | - | [M+H] | 46.81 | - |
| 74 | RT35.895 | - | - | 57 | 35.895 | - | - | 2111.4 | - | - | 6418.57143 | - | | - | - | [M+H] | 6.76 | - |
| 75 | RT36.240 | - | - | 73 | 36.240 | - | - | 2148.6 | - | - | 35663 | - | | - | - | [M+H] | 6.39 | - |
| 76 | RT39.154 | - | - | 73 | 39.154 | - | - | 2484 | - | - | 1077007.57 | - | | - | - | [M+H] | 4.32 | - |
| 78 | RT41.145 | - | - | 73 | 41.145 | - | - | 2740.3 | - | - | 377977 | - | | - | - | [M+H] | 1.5 | - |

**Table S2.** Volatile organic compounds experimentally obtained in Hass avocado pulp.

| # | Compound | Common name | Formula | m/z | RT | Exact Mass | BP (°C) | Exp KI | Theo KI | SI | Average area | %Error KI | Pubchem CID | HMDB | Adduct | S/N | Odor |
| --- | --- | --- | --- | --- | --- | --- | --- | --- | --- | --- | --- | --- | --- | --- | --- | --- | --- |
| 1 | Acetone | Acetone | C_3_H_6_O | 43 | 2.004 | 58.0418 | 56.08 | - | 475.3 | 97 | 1783361.286 | - | 180 | HMDB0001659 | [M+H] | 18.45 | - |
| 2 | RT2.085 | - | - | 43 | 2.085 | - | - | - | - | - | 299827.4286 | - | - | - | [M+H] | 13.86 | - |
| 3 | RT2.325 | - | - | 75 | 2.325 | - | - | 602.01 | - | - | 603007.8571 | - | - | - | [M+H] | 6.24 | - |
| 4 | RT2.530 | - | - | 43 | 2.530 | - | - | 629.53 | - | - | 1151344.571 | - | - | - | [M+H] | 6.5 | - |
| 5 | RT3.150 | - | - | 43 | 3.150 | - | - | 703.68 | - | - | 281725.1429 | - | - | - | [M+H] | 17.14 | - |
| 6 | 2,27 Pentanal | Pentanal | C_5_H_10_O | 44 | 3.400 | 86.0731 | 103 | 713.35 | 674 | 92 | 214950.1429 | 5.83771 | 8063 | HMDB0031206 | [M+H] | 1.82 | almond, malt, pungent |
| 7 | RT3.630 | - | - | 96 | 3.630 | - | - | 722.24 | - | - | 456428 | - | - | - | [M+H] | 4.66 | - |
| 8 | RT4.100 | - | - | 73 | 4.100 | - | - | 740.43 | - | - | 20889.14286 | - | - | - | [M+H] | 0.79 | - |
| 9 | RT4.845 | - | - | 91 | 4.845 | - | - | 769.25 | - | - | 235283.8571 | - | - | - | [M+H] | 23.05 | - |
| 10 | RT5.285 | - | - | 55 | 5.285 | - | - | 786.27 | - | - | 338729.7143 | - | - | - | [M+H] | 3.38 |  |
| 11 | RT5.325 | - | - | 55 | 5.325 | - | - | 787.81 | - | - | 127128.5714 | - | - | - | [M+H] | 1.68 |  |
| 12 | Hexanal | Hexanal | C_6_H_12_O | 44 | 5.424 | 100.088 | 129,6 | 791.64 | 769 | 99 | 148964.5714 | 2.94461 | 6184 | HMDB05994 | [M+H] | 36.62 | grass, tallow, fat |
| 13 | RT5.545 | - | - | 55 | 5.545 | - | - | 796.32 | - | - | 155967.8571 | - | - | - | [M+H] | 4.94 | - |
| 14 | RT5.730 | - | - | 207 | 5.730 | - | - | 802.87 | - | - | 2355.857143 | - | - | - | [M+H] | 0.39 | - |
| 15 | RT5.835 | - | - | 55 | 5.835 | - | - | 806.22 | - | - | 652837.5714 | - | - | - | [M+H] | 2.1 | - |
| 16 | RT6.030 | - | - | 207 | 6.030 | - | - | 812.44 | - | - | 145325.7143 | - | - | - | [M+H] | 3.36 | - |
| 17 | RT7.085 | - | - | 41 | 7.085 | - | - | 846.09 | - | - | 12944.85714 | - | - | - | [M+H] | 3.95 | - |
| 18 | 2-Hexenal | 2-Hexenal | C_6_H_10_O | 41 | 7.370 | 98.0731 | 47 | 855.18 | 820 | 98 | 98769.28571 | 4.29066 | 5281168 | HMDB31496 | [M+H] | 30.72 | apple, green, leaf, fat, rancid |
| 19 | RT7.970 | - | - | 133 | 7.970 | - | - | 874.32 | - | - | 23796.57143 | - | - | - | [M+H] | 0.92 | - |
| 20 | Cyclohexanone | Cyclohexanone | C_6_H_10_O | 42 | 8.960 | 98.0731 | 155,4 | 904.42 | 851.7 | 93 | 64909.14286 | 6.18941 | 7967 | HMDB0003315 | [M+H] | 10.72 | - |
| 21 | RT10.785 | - | - | 281 | 10.785 | - | - | 947.97 | - | - | 26417 | - | - | - | [M+H] | 2.18 | - |
| 22 | RT10.860 | - | - | 281 | 10.860 | - | - | 949.76 | - | - | 4664.285714 | - | - | - | [M+H] | 1.68 | - |
| 23 | RT11.710 | - | - | 282 | 11.710 | - | - | 970.05 | - | - | 45466 | - | - | - | [M+H] | 0.84 | - |
| 24 | RT11.855 | - | - | 207 | 11.855 | - | - | 973.51 | - | - | 104496.8571 | - | - | - | [M+H] | 1.01 | - |
| 25 | RT13.340 | - | - | 41 | 13.340 | - | - | 1008 | - | - | 1843.571429 | - | - | - | [M+H] | 3.48 | - |
| 26 | RT13.870 | - | - | 79 | 13.870 | - | - | 1019.4 | - | - | 17453.57143 | - | - | - | [M+H] | 6.19 | - |
| 27 | RT15.650 | - | - | 73 | 15.650 | - | - | 1057.5 | - | - | 6724.428571 | - | - | - | [M+H] | 23.15 | - |
| 28 | RT17.250 | - | - | 53 | 17.250 | - | - | 1091.8 | - | - | 3673.857143 | - | - | - | [M+H] | 1.2 | - |
| 29 | RT17.540 | - | - | 117 | 17.540 | - | - | 1098 | - | - | 11992.28571 | - | - | - | [M+H] | 4.01 | - |
| 30 | RT17.935 | - | - | 41 | 17.935 | - | - | 1106.3 | - | - | 284338.2857 | - | - | - | [M+H] | 2.06 | - |
| 31 | Nonanal | Nonanal | C_9_H_18_O | 41 | 18.140 | 142.135 | 195 | 1110.6 | 1081 | 93 | 69710.71429 | 2.74112 | 31289 | HMDB0059835 | [M+H] | 9.89 | fat, citrus, green |
| 32 | RT18.720 | - | - | 41 | 18.720 | - | - | 1122.8 | - | - | 193200.5714 | - | - | - | [M+H] | 1.67 | - |
| 33 | RT19.470 | - | - | 79 | 19.470 | - | - | 1138.6 | - | - | 92516.14286 | - | - | - | [M+H] | 2.82 | - |
| 34 | RT19.675 | - | - | 91 | 19.675 | - | - | 1142.9 | - | - | 2012.857143 | - | - | - | [M+H] | 2.02 | - |
| 35 | RT20.205 | - | - | 73 | 20.205 | - | - | 1154.1 | - | - | 3136.857143 | - | - | - | [M+H] | 2.23 | - |
| 36 | 2-Tridecene, (Z)- | cis-2-Tridecene | C_13_H_26_ | 41 | 22.705 | 182.203 | - | 1210.8 | 1311 | 93 | 41947 | 7.64497 | 5362714 | - | [M+H] | 4.12 | - |
| 37 | RT23.185 | - | - | 41 | 23.185 | - | - | 1226.9 | - | - | 7058 | - | - | - | [M+H] | 1.44 | - |
| 38 | RT24.010 | - | - | 117 | 24.010 | - | - | 1254.7 | - | - | 2302.571429 | - | - | - | [M+H] | 4.29 | - |
| 39 | RT24.270 | - | - | 44 | 24.270 | - | - | 1263.5 | - | - | 7576.857143 | - | - | - | [M+H] | 12.83 | - |
| 40 | RT25.500 | - | - | 73 | 25.500 | - | - | 1307.2 | - | - | 17269.28571 | - | - | - | [M+H] | 19.21 | - |
| 41 | ,alpha,-ylangene | alpha-Ylangene | C_15_H_24_ | 105 | 26.940 | 204.187 | - | 1379.1 | - | 99 | 4068.571429 | - | 442409 | - | [M+H] | 20.07 | - |
| 42 | RT28.005 | - | - | 41 | 28.005 | - | - | 1440.3 | - | - | 25692.28571 | - | - | - | [M+H] | 3.17 | - |
| 43 | trans-.alpha.-Bergamotene | trans-alpha-Bergamotene | C_15_H_24_ | 93 | 28.050 | 204.187 | - | 1443.1 | 1496 | 99 | 181885 | 3.53442 | 6429302 | - | [M+H] | 35.22 | wood, warm, tea |
| 44 | Caryophyllene <(Z)-> | Caryophyllene | C_15_H_24_ | 41 | 28.335 | 204.187 | - | 1460.9 | - | 100 | 2878.714286 | - | 5498518 | HMDB0036792 | [M+H] | 9.77 | - |
| 45 | RT28.797 | - | - | 341 | 28.797 | - | - | 1489.8 | - | - | 6098 | - | - | - | [M+H] | 3.53 | - |
| 46 | RT29.120 | - | - | 81 | 29.120 | - | - | 1511.6 | - | - | 18623.42857 | - | - | - | [M+H] | 7.04 | - |
| 47 | RT29.545 | - | - | 41 | 29.545 | - | - | 1542.5 | - | - | 27530.85714 | - | - | - | [M+H] | 3.43 | - |
| 48 | RT30.685 | - | - | 416 | 30.685 | - | - | 1628.3 | - | - | 4040.428571 | - | - | - | [M+H] | 1.47 | - |
| 49 | RT30.860 | - | - | 167 | 30.860 | - | - | 1642.5 | - | - | 18589.42857 | - | - | - | [M+H] | 9.74 | - |
| 50 | Benzene, undecyl- | Undecylbenzene | C_17_H_28_ | 92 | 31.730 | 232.219 | 316 | 1714 | 1760 | 99 | 587435.8571 | 2.62176 | 23194 | - | [M+H] | 168.6 | - |
| 51 | 1-Pentadecene | 1-Pentadecene | C_15_H_30_ | 43 | 31.820 | 210.234 | - | 1721.8 | 1486 | 96 | 45270.71429 | 15.8703* | 25913 | HMDB0031082 | [M+H] | 9.03 | - |
| 52 | exo-7-(trans-1-Propenyl)bicyclo[4,2,0]oct-1(2)-ene | 7-[(1E)-1-Propenyl]bicyclo[4.2.0]oct-1-ene | C_11_H_16_ | 91 | 32.290 | 148.125 | - | 1762.9 | - | 91 | 411806 | - | 5368398 | - | [M+H] | 2.65 | - |
| 53 | RT33.575 | - | - | 149 | 33.575 | - | - | 1879.6 | - | - | 12161 | - | - | - | [M+H] | 6.91 | - |
| 54 | cis-8-Isopropylbicyclo[4,3,0]non-3-ene | 2-Isopropyl-2,3,3a,4,7,7a-hexahydro-1H-inden | C_12_H_20_ | 121 | 33.920 | 164.156 | - | 1912.3 | - | 93 | 870432.4286 | - | 578143 | - | [M+H] | 32.61 | - |
| 55 | Benzene, dodecyl- | Dodecylbenzene | C_18_H_30_ | 92 | 34.055 | 246.234 | 328 | 1925.5 | 1866 | 98 | 9344747.286 | 3.18811 | 31237 | HMDB0251572 | [M+H] | 118.5 | - |
| 56 | 1-Heptadec-1-ynyl-cyclopentanol | 1-(1-Heptadecynyl)cyclopentanol | C_22_H_40_O | 111 | 34.145 | 320.307 | - | 1934.3 | - | 98 | 16334124.57 | - | 536340 | - | [M+H] | 58.93 | - |
| 57 | Indane, 1-nonyl- | 1-Nonylindane | C_18_H_28_ | 117 | 34.420 | 244.219 | - | 1961.3 | - | 99 | 3381783.286 | - | 575844 | - | [M+H] | 92.79 | - |
| 58 | Naphthalene, 1,2,3,4-tetrahydro-1-octyl- | 1-Octyl-1,2,3,4-tetrahydronaphthalene | C_18_H_28_ | 131 | 34.610 | 244.219 | - | 1979.9 | - | 98 | 1480880.143 | - | 583061 | - | [M+H] | 51.48 | - |
| 59 | Hexadecanoic acid <n-> | palmitic acid | C_16_H_32_O_2_ | 43 | 34.944 | 256.240 | 351,5 | 2013.2 | 1942 | 98 | 47551740.71 | 3.66790 | 985 | - | [M+H] | 40.75 | - |
| 60 | 9-Hexadecenoic acid | (E)-hexadec-9-enoic acid | C_16_H_30_O_2_ | 55 | 36.731 | 254.224 | 363 | 2201.8 | 1916 | 94 | 6670561.143 | 14.8984 | 5282745 | - | [M+H] | 8.58 | - |
| 61 | Adamantane, 1,3-dimethyl- | 1-3-Dimethyladamantane | C_12_H_20_ | 149 | 37.530 | 164.156 | - | 2291.1 | 1184 | 97 | 1252014 | 93.5018* | 12800 | - | [M+H] | 73.55 | - |
| 62 | RT37.767 | - | - | 73 | 37.767 | - | - | 2318.4 | - | - | 59876 | - | - | - | [M+H] | 30.25 | - |
| 63 | 6-Pentadecanone | 6-Pentadecanone | C_15_H_30_O | 43 | 37.770 | 226.229 | - | 2318.7 | - | 96 | 1054455.857 | - | 70476 | - | [M+H] | 3.3 | - |
| 64 | 2(3H)-Furanone, dihydro-5-tetradecyl- | gamma-Stearolactone | C_18_H_34_O_2_ | 85 | 38.147 | 282.255 | - | 2362.8 | - | 99 | 1554127.143 | - | 10396 | - | [M+H] | 24.24 | - |
| 65 | RT38.465 | - | - | 99 | 38.465 | - | - | 2400 | - | - | 648651.1429 | - | - | - | [M+H] | 18.26 | - |
| 66 | Heptadecane | Heptadecane | C_17_H_36_ | 57 | 38.720 | 240.281 |  | 2431.1 | 1700 | 94 | 499574.8571 | 43.0057* | 12398 | HMDB0059830 | [M+H] | 3.85 | - |
| 67 | RT39.154 | - | - | 73 | 39.154 | - | - | 2484 | - | - | 1144890.286 | - | - | - | [M+H] | 8.77 | - |
| 68 | RT39.250 | - | - | 43 | 39.250 | - | - | 2495.7 | - | - | 217424.2857 | - | - | - | [M+H] | 1.82 | - |
| 69 | RT39.955 | - | - | 73 | 39.955 | - | - | 2584.3 | - | - | 280485.4286 | - | - | - | [M+H] | 4.43 | - |
| 70 | 2-methyloctacosane | 2-Methyloctacosane | C_29_H_60_ | 57 | 40.916 | 408.469 | 414 | 2709.5 | 2862 | 98 | 469343.8571 | 5.32738 | 519147 | HMDB0029701 | [M+H] | 14.72 | - |
| 71 | Docosane | Docosane | C_22_H_46_ | 57 | 41.165 | 310.359 | 369 | 2743 | 2200 | 97 | 1627223.714 | 24.6796 | 12405 | HMDB0061865 | [M+H] | 27.63 | - |

**Table S3.** Volatile organic compounds experimentally obtained in Hass avocado seed.

| # | Compound | Common name | Formula | m/z | RT | Exact Mass | BP (°C) | Exp KI | Theo KI | SI | Average area | %Error KI | Pubchem CID | HMDB | Adduct | S/N | Odor |
| --- | --- | --- | --- | --- | --- | --- | --- | --- | --- | --- | --- | --- | --- | --- | --- | --- | --- |
| 1 | Acetone | Acetone | C_3_H_6_O | 43 | 2.004 | 58.04186 | 56.08 | - | - | 94 | 403413.5714 | - | 180 | HMDB0001659 | [M+H] | 23.71 | - |
| 2 | RT2.015 | - | - | 43 | 2.015 | - | - | - | - | - | 967990 | - | - | - | [M+H] | 23.82 | - |
| 3 | RT2.530 | - | - | 43 | 2.530 | - | - | 629.53 | - | - | 637469.4286 | - | - | - | [M+H] | 14.43 | - |
| 4 | Cyclopentene, 4-methyl- | 4-Methylcyclopentene. | C_6_H_10_ | 67 | 2.890 | 82.07825 | - | 677.85 | 618.7 | 92 | 119526.1429 | 9.5607 | 15658 | - | [M+H] | 42.96 | - |
| 5 | RT3.000 | - | - | 43 | 3.000 | - | - | 692.62 | - | - | 217159.5714 | - | - | - | [M+H] | 0.78 | - |
| 6 | RT3.150 | - | - | 43 | 3.150 | - | - | 703.68 | - | - | 204041.4286 | - | - | - | [M+H] | 33.5 | - |
| 7 | RT4.380 | - | - | 81 | 4.380 | - | - | 751.26 | - | - | 4824.428571 | - | - | - | [M+H] | 7.46 | - |
| 8 | Hexanal | Hexanal | C_6_H_12_O | 44 | 5.424 | 100.0888 | 129.6 | 791.64 | 769 | 100 | 1070309.286 | 2.9446 | 6184 | HMDB05994 | [M+H] | 365.9 | grass, tallow, fat |
| 9 | RT5.920 | - | - | 207 | 5.920 | - | - | 808.93 | - | - | 440673.7143 | - | - | - | [M+H] | 10.03 | - |
| 10 | RT8.420 | - | - | 43 | 8.420 | - | - | 888.68 | - | - | 5496.571429 | - | - | - | [M+H] | 6.23 | - |
| 11 | RT8.555 | - | - | 107 | 8.555 | - | - | 892.98 | - | - | 4269.857143 | - | - | - | [M+H] | 9.61 | - |
| 12 | RT9.375 | - | - | 43 | 9.375 | - | - | 914.32 | - | - | 49104.57143 | - | - | - | [M+H] | 9.47 | - |
| 13 | RT9.995 | - | - | 93 | 9.995 | - | - | 929.12 | - | - | 1807.142857 | - | - | - | [M+H] | 6.61 | - |
| 14 | (1S)-2,6,6-Trimethylbicyclo[3,1,1]hept-2-ene | alpha.-Pinene | C_10_H_16_ | 93 | 10.335 | 136.1252 | - | 937.23 | 922.6 | 90 | 216727.4286 | 1.5892 | 12223113 | HMDB0302508 | [M+H] | 26.93 | pine, turpentine |
| 15 | Camphene | Camphene | C_10_H_16_ | 93 | 11.125 | 136.1252 | 161 | 956.09 | 943 | 94 | 293102.2857 | 1.3876 | 6616 | HMDB0059839 | [M+H] | 6.94 | camphor |
| 16 | RT11.270 | - | - | 281 | 11.270 | - | - | 959.55 | - | - | 158367.4286 | - | - | - | [M+H] | 1.1 | - |
| 17 | ,beta,-Pinene | (+)-beta-Pinene | C_10_H_16_ | 93 | 12.015 | 136.1252 | - | 977.33 | - | 98 | 205071.8571 | - | 10290825 | - | [M+H] | 427.3 | pine, resin, turpentine |
| 18 | Myrcene | Myrcene | C_10_H_16_ | 41 | 12.590 | 136.1252 | 167 | 991.05 | 979 | 98 | 440107.8571 | 1.2308 | 31253 | HMDB0038169 | [M+H] | 698.2 | balsamic, must, spice |
| 19 | RT12.745 | - | - | 60 | 12.745 | - | - | 994.75 | - | - | 158383.8571 | - | - | - | [M+H] | 1.15 | - |
| 20 | RT13.545 | - | - | 93 | 13.545 | - | - | 1012.4 | - | - | 12383.57143 | - | - | - | [M+H] | 0.19 | - |
| 21 | Carene<delta-2-> | (+)-2-Carene | C_10_H_16_ | 93 | 13.780 | 136.1252 | - | 1017.5 | - | 97 | 142598 | - | 78249 | - | [M+H] | 26.71 | - |
| 22 | trans-3-Caren-2-ol | trans-3-Caren-2-ol | C_10_H_16_O | 119 | 14.233 | 152.1201 | - | 1027.2 | 1198 | 91 | 48177 | 14.261 | 576906 | - | [M+H] | 12.46 | - |
| 23 | Terpinolene | alpha-Terpinolene | C_10_H_16_ | 93 | 14.250 | 136.1252 | 187 | 1027.5 | 1078 | 99 | 105299.7143 | 4.6831 | 11463 | HMDB0036994 | [M+H] | 48.71 | - |
| 24 | Limonene | Limonene | C_10_H_16_ | 68 | 14.425 | 136.1252 | 177.8 | 1031.3 | 1020 | 94 | 62713 | 1.1042 | 22311 | HMDB0032473 | [M+H] | 22.8 | lemon, orange, citrus, mint |
| 25 | RT14.430 | - | - | 119 | 14.430 | - | - | 1031.4 | - | - | 4436.571429 | - | - | - | [M+H] | 6.1 | - |
| 26 | p-Cymene | p-Cymene | C_10_H_14_ | 119 | 14.655 | 134.1095 | 177.1 | 1036.2 | 1011 | 97 | 106051.5714 | 2.4914 | 7463 | HMDB0005805 | [M+H] | 35.66 | solvent, gasoline, citrus |
| 27 | \|E\|-_b_-Ocimene | (E)-beta-ocimene | C_10_H_16_ | 93 | 15.285 | 136.1252 | 174 | 1049.7 | 1041 | 98 | 77977.42857 | 0.8336 | 5281553 | HMDB0030089 | [M+H] | 140.2 | sweet, herb |
| 28 | RT15.560 | - | - | 43 | 15.560 | - | - | 1055.6 | - | - | 4802.142857 | - | - | - | [M+H] | 4.1 | - |
| 29 | Carene<delta-3-> | Delta-3-Carene | C_10_H_16_ | 93 | 15.748 | 136.1252 | 174 | 1059.6 | - | 98 | 42845.85714 | - | 26049 | HMDB0035619 | [M+H] | 7.3 | lemon, resin |
| 30 | Terpinene<gamma-> | Gamma-terpinene | C_10_H_16_ | 93 | 16.225 | 136.1252 | 183 | 1069.8 | 1047 | 96 | 26060.14286 | 2.1783 | 7461 | HMDB0005806 | [M+H] | 40.42 | gasoline, turpentine |
| 31 | RT16.865 | - | - | 79 | 16.865 | - | - | 1083.5 | - | - | 9259.857143 | - | - | - | [M+H] | 4.87 | - |
| 32 | RT17.200 | - | - | 93 | 17.200 | - | - | 1090.7 | - | - | 5286.428571 | - | - | - | [M+H] | 1.48 | - |
| 33 | Cyclohexene, 1-methyl-4-(1-methylethylidene)- | Terpinolene | C_10_H_16_ | 93 | 17.560 | 136.1252 | 187 | 1098.3 | 1078 | 97 | 175613.8571 | 1.8918 | 11463 | HMDB0036994 | [M+H] | 55.47 | - |
| 34 | Benzene, 1,2,3,4-tetramethyl- | 1,2,3,4-tetramethylbenzene | C_10_H_14_ | 119 | 19.175 | 134.1095 | - | 1132.4 | 1145.3 | 90 | 13356.42857 | 1.1245 | 10263 | HMDB0059823 | [M+H] | 28.66 | - |
| 35 | RT20.395 | - | - | 91 | 20.395 | - | - | 1158.1 | - | - | 12511.42857 | - | - | - | [M+H] | 1.63 | - |
| 36 | RT20.510 | - | - | 44 | 20.510 | - | - | 1160.5 | - | - | 3253.428571 | - | - | - | [M+H] | 10.7 | - |
| 37 | RT20.655 | - | - | 119 | 20.655 | - | - | 1163.5 | - | - | 17389 | - | - | - | [M+H] | 4.97 | - |
| 38 | RT21.190 | - | - | 104 | 21.190 | - | - | 1174.8 | - | - | 6557.857143 | - | - | - | [M+H] | 12.04 | - |
| 39 | RT22.545 | - | - | 43 | 22.545 | - | - | 1205.3 | - | - | 9242.857143 | - | - | - | [M+H] | 0.35 | - |
| 40 | 1H-Indene, 2,3-dihydro-1,2-dimethyl- | 1,2-Dimethylindane | C_11_H_14_ | 131 | 22.770 | 146.1095 | - | 1212.9 | 1130.1 | 95 | 54337.57143 | 7.3323 | 28225 | - | [M+H] | 47.69 | - |
| 41 | RT23.185 | - | - | 55 | 23.185 | - | - | 1226.9 | - | - | 11625.57143 | - | - | - | [M+H] | 2.35 | - |
| 42 | RT23.270 | - | - | 43 | 23.270 | - | - | 1229.7 | - | - | 10377.14286 | - | - | - | [M+H] | 5.04 | - |
| 43 | RT24.430 | - | - | 73 | 24.430 | - | - | 1268.8 | - | - | 108338.5714 | - | - | - | [M+H] | 6.09 | - |
| 44 | RT24.545 | - | - | 73 | 24.545 | - | - | 1272.7 | - | - | 32982.85714 | - | - | - | [M+H] | 0.68 | - |
| 45 | RT24.705 | - | - | 41 | 24.705 | - | - | 1278.1 | - | - | 24808.28571 | - | - | - | [M+H] | 2.94 | - |
| 46 | RT24.800 | - | - | 131 | 24.800 | - | - | 1281.3 | - | - | 49597 | - | - | - | [M+H] | 1.38 | - |
| 47 | RT25.300 | - | - | 43 | 25.300 | - | - | 1298.1 | - | - | 15255.71429 | - | - | - | [M+H] | 1.21 | - |
| 48 | RT25.395 | - | - | 73 | 25.395 | - | - | 1301.9 | - | - | 13047.71429 | - | - | - | [M+H] | 1.23 | - |
| 49 | 20,18 Undec-10-en-1-al | 10-Undecenal | C_11_H_20_O | 41 | 25.598 | 168.1514 | 100 | 1312.1 | 1277.2 | 98 | 25278.71429 | 2.7340 | 8187 | HMDB0031128 | [M+H] | 1.04 | - |
| 50 | RT25.975 | - | - | 105 | 25.975 | - | - | 1330.9 | - | - | 55342.14286 | - | - | - | [M+H] | 2.46 | - |
| 51 | RT26.675 | - | - | 161 | 26.675 | - | - | 1365.8 | - | - | 76260.42857 | - | - | - | [M+H] | 1.16 | - |
| 52 | RT26.705 | - | - | 91 | 26.705 | - | - | 1367.3 | - | - | 63343 | - | - | - | [M+H] | 2.51 | - |
| 53 | RT26.760 | - | - | 133 | 26.760 | - | - | 1370.0 | - | - | 110580.2857 | - | - | - | [M+H] | 1.01 | - |
| 54 | ,alpha,-ylangene | alpha-Ylangene | C_15_H_24_ | 105 | 26.940 | 204.1878 | - | 1379.0 | - | 96 | 825374.4286 | - | 442409 | - | [M+H] | 4.18 | - |
| 55 | Copaene <alpha-> | (-)-alpha-Copaene | C_15_H_24_ | 119 | 27.060 | 204.1878 | - | 1385.0 | - | 99 | 900823.8571 | - | 12303902 | HMDB0061851 | [M+H] | 4.58 | wood, spice |
| 56 | Aromadendrene | (-)-Aromadendrene | C_15_H_24_ | 93 | 27.155 | 204.1878 | 121 | 1389.7 | 1439 | 98 | 57044.14286 | 3.4207 | 91354 | HMDB0036418 | [M+H] | 4.74 | wood |
| 57 | Eremophilene | Eremophilene | C_15_H_24_ | 105 | 27.155 | 204.1878 | - | 1389.7 | 1512 | 98 | 217286.7143 | 8.0836 | 12309744 | - | [M+H] | 1.64 | - |
| 58 | RT27.310 | - | - | 189 | 27.310 | - | - | 1397.5 | - | - | 345559.4286 | - | - | - | [M+H] | 9.32 | - |
| 59 | RT27.835 | - | - | 204 | 27.835 | - | - | 1429.6 | - | - | 245276.1429 | - | - | - | [M+H] | 8.74 | - |
| 60 | Maaliene <beta-> | beta-Maaliene | C_15_H_24_ | 161 | 28.555 | 204.1878 | - | 1474.6 | - | 95 | 5020668.571 | - | 521242 | - | [M+H] | 2.83 | - |
| 61 | RT28.695 | - | - | 41 | 28.695 | - | - | 1483.4 | - | - | 89663.57143 | - | - | - | [M+H] | 1.48 | - |
| 62 | \|cis\|-_b_-Guaiene | cis-beta-Guaiene | C_15_H_24_ | 161 | 28.715 | 204.1878 | - | 1484.6 | 1478 | 94 | 2614656.286 | 0.4524 | 15560253 | - | [M+H] | 1.57 | wood, spice |
| 63 | Naphthalene, 1,2,4a,5,6,8a-hexahydro-4,7-dimethyl-1-(1-methylethyl)- | .alpha.-Amorphene | C_15_H_24_ | 105 | 28.775 | 204.1878 | - | 1488.4 | 1479 | 97 | 2479830.143 | 0.6381 | 101708 | HMDB0037784 | [M+H] | 3.23 | - |
| 64 | 29,29 Amorphene<delta-> | (+)-delta-amorphene | C_15_H_24_ | 105 | 28.825 | 204.1878 | - | 1491.5 | - | 95 | 75673.71429 | - | 10223 | HMDB0030644 | [M+H] | 0.93 | - |
| 65 | 26,78 Muurola-3,5-diene<trans-> | trans-Muurola-4(14),5-diene | C_15_H_24_ | 105 | 28.995 | 204.1878 | - | 1502.5 | 1454 | 91 | 35247 | 3.3387 | 91747125 | HMDB0061841 | [M+H] | 1.67 | - |
| 66 | 28,49 Selinene<delta-> | delta-Selinene | C_15_H_24_ | 161 | 29.070 | 204.1878 | - | 1508 | 1509 | 94 | 203339.2857 | 0.0662 | 520383 | - | [M+H] | 2.8 | - |
| 67 | Epizonarene | Epizonarene | C_15_H_24_ | 161 | 29.180 | 204.1878 | - | 1516 | 1499 | 97 | 11037602 | 1.1340 | 595385 | - | [M+H] | 1.98 | - |
| 68 | RT29.215 | - | - | 41 | 29.215 | - | - | 1518.5 | - | - | 180304.4286 | - | - | - | [M+H] | 0.99 | - |
| 69 | RT29.330 | - | - | 161 | 29.330 | - | - | 1538.1 | - | - | 160912.7143 | - | - | - | [M+H] | 1.73 | - |
| 70 | Cadinene <delta-> | (+)-delta-Cadinene | C_15_H_24_ | 161 | 29.413 | 204.1878 | 121 | 1540 | - | 98 | 8919.857143 | - | 441005 | HMDB0035084 | [M+H] | 1.93 | thyme, medicine, wood |
| 71 | _w_-Cadinene | omega-Cadinene | C_15_H_24_ | 119 | 29.530 | 204.1878 | - | 1541.4 | - | 99 | 94307 | - | 10375655 | HMDB0038203 | [M+H] | 5.67 | - |
| 72 | RT29.545 | - | - | 145 | 29.545 | - | - | 1542.5 | - | - | 106963.1429 | - | - | - | [M+H] | 7.97 | - |
| 73 | _a_-Cubebene | (-)-alpha-Cubebene | C_15_H_24_ | 119 | 29.675 | 204.1878 | 246 | 1552 | - | 91 | 111394.2857 | - | 442359 | HMDB0036413 | [M+H] | 4.05 | herb, wax |
| 74 | Isocalamenene | Isocalamenene | C_15_H_22_ | 159 | 29.705 | 202.1721 | - | 1554.1 | 1524 | 99 | 268825.8571 | 1.9804 | 15559873 | - | [M+H] | 38.75 | - |
| 75 | RT29.820 | - | - | 119 | 29.820 | - | - | 1564.7 | - | - | 7373.857143 | - | - | - | [M+H] | 1.46 | - |
| 76 | RT30.165 | - | - | 43 | 30.165 | - | - | 1587.6 | - | - | 210523.5714 | - | - | - | [M+H] | 1.12 | - |
| 77 | RT30.580 | - | - | 131 | 30.580 | - | - | 1619.8 | - | - | 89870.28571 | - | - | - | [M+H] | 7.97 | - |
| 78 | RT30.720 | - | - | 105 | 30.720 | - | - | 1631.1 | - | - | 88720 | - | - | - | [M+H] | 2.43 | - |
| 79 | RT31.380 | - | - | 43 | 31.380 | - | - | 1684.1 | - | - | 2831.428571 | - | - | - | [M+H] | 0.6 | - |
| 80 | Benzene, undecyl- | Undecylbenzene | C_17_H_28_ | 92 | 31.730 | 232.2191 | 316 | 1713.9 | 1760 | 99 | 892579.5714 | 2.6217 | 23194 | - | [M+H] | 137.6 | - |
| 81 | RT31.925 | - | - | 43 | 31.925 | - | - | 1731.0 | - | - | 295148.7143 | - | - | - | [M+H] | 2.82 | - |
| 82 | exo-7-(trans-1-Propenyl)bicyclo[4,2,0]oct-1(2)-ene | 7-[(1E)-1-Propenyl]bicyclo[4.2.0]oct-1-ene | C_11_H_16_ | 91 | 32.290 | 148.1252 | - | 1762.8 | - | 94 | 630695.4286 | - | 5368398 | - | [M+H] | 3.59 | - |
| 83 | RT32.750 | - | - | 73 | 32.750 | - | - | 1803.2 | - | - | 619370 | - | - | - | [M+H] | 1.83 | - |
| 84 | RT33.605 | - | - | 121 | 33.605 | - | - | 1882.4 | - | - | 2444506.286 | - | - | - | [M+H] | 11.23 | - |
| 85 | RT33.705 | - | - | 94 | 33.705 | - | - | 1891.6 | - | - | 261432.8571 | - | - | - | [M+H] | 7.04 | - |
| 86 | Benzene, dodecyl- | Dodecylbenzene | C_18_H_30_ | 92 | 34.055 | 246.2347 | 328 | 1925.4 | 1866 | 99 | 8198791.571 | 3.1881 | 31237 | HMDB0251572 | [M+H] | 191.2 | - |
| 87 | 1-Heptadec-1-ynyl-cyclopentanol | 1-(1-Heptadecynyl)cyclopentanol | C_22_H_40_O | 111 | 34.145 | 320.3079 | - | 1934.3 | - | 98 | 7514671.714 | - | 536340 | - | [M+H] | 58.32 | - |
| 88 | RT34.400 | - | - | 117 | 34.400 | - | - | 1959.3 | - | - | 2827071.429 | - | - | - | [M+H] | 129.0 | - |
| 89 | Indane, 1-nonyl- | 1-Nonylindane | C_18_H_28_ | 117 | 34.420 | 244.2191 | - | 1961.2 | - | 97 | 3701110.429 | - | 575844 | - | [M+H] | 125.2 | - |
| 90 | RT34.604 | - | - | 131 | 34.604 | - | - | 1979.3 | - | - | 2097153.429 | - | - | - | [M+H] | 62 | - |
| 91 | RT35.570 | - | - | 41 | 35.570 | - | - | 2077.4 | - | - | 5253935 | - | - | - | [M+H] | 0.79 | - |
| 92 | RT37.820 | - | - | 73 | 37.820 | - | - | 2324.5 | - | - | 633081.5714 | - | - | - | [M+H] | 5.47 | - |
| 93 | 2(3H)-Furanone, dihydro-5-tetradecyl- | gamma-Stearolactone | C_18_H_34_O_2_ | 85 | 38.147 | 282.2558 | - | 2362.8 | - | 98 | 1056336 | - | 10396 | - | [M+H] | 67.88 | - |
| 94 | RT39.545 | - | - | 41 | 39.545 | - | - | 2532.7 | - | - | 290919.7143 | - | - | - | [M+H] | 25.72 | - |
| 95 | Hexacosane | Hexacosane | C_26_H_54_ | 57 | 39.591 | 366.4225 | 415 | 2538.4 | 2600 | 91 | 1007981.571 | 2.3657 | 12407 |  | [M+H] | 69.92 | - |
| 96 | 2-methyloctacosane | 2-Methyloctacosane | C_29_H_60_ | 57 | 40.916 | 408.4695 | 414 | 2709.5 | 2862 | 97 | 777830.5714 | 5.3273 | 519147 | - | [M+H] | 66.9 | - |
